# Controlled Secondary Growth of CAU-1-NH_2_ Membranes with Improved CO_2_ Separation Performance

**DOI:** 10.1021/acs.langmuir.6c02353

**Published:** 2026-07-03

**Authors:** Bing-Han Lin, Chia-Hui Chuang, Li-Wei Hsiao, Hsiang-Yu Wang, Li-Tang Chi, Yi-Hsuan Lin, Li-Chiang Lin, Dun-Yen Kang

**Affiliations:** Department of Chemical Engineering, 33561 National Taiwan University, No. 1, Sec. 4, Roosevelt Road, Taipei 10617, Taiwan

## Abstract

Metal–organic framework (MOF) membranes have attracted increasing interest for energy-efficient gas separation due to their tunable pore structures and selective adsorption properties. In this study, CAU-1-NH_2_ membranes were fabricated on porous α-alumina substrates via a seeded growth method. The effects of precursor concentration and ligand-to-metal ratio in the secondary growth solution on membrane morphology and gas separation performance were systematically investigated. Among the five membrane variants synthesized, the optimized CAU-1-NH_2_(B) membrane exhibited a continuous and low-defect structure, as confirmed by scanning electron microscopy and confocal fluorescence microscopy. Single-gas permeation measurements on the CAU-1-NH_2_(B) membrane at 35 °C and 3 bar showed H_2_, CO_2_, N_2_, and CH_4_ permeances of 207.1, 110.2, 6.0, and 6.6 GPU, respectively, corresponding to ideal CO_2_/N_2_ and CO_2_/CH_4_ selectivities of 19.4 and 17.6. Mixed-gas permeation tests revealed significantly enhanced separation performance, with CO_2_/N_2_ separation factors ranging from 59.3 to 89.2 under various feed compositions. Grand canonical Monte Carlo (GCMC) simulations further indicate that strong competitive adsorption of CO_2_ within the CAU-1-NH_2_ framework plays a dominant role in governing the observed mixed-gas separation behavior. These findings demonstrate the potential of CAU-1-NH_2_ membranes for efficient CO_2_ separation.

## Graphical abstract

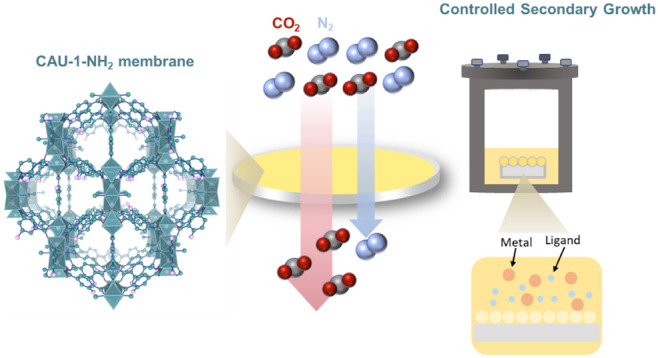

## Introduction

Metal–organic frameworks (MOFs) are porous crystalline materials constructed from metal ions or clusters coordinated with organic ligands. In recent years, MOFs have garnered increasing attention in materials science due to their exceptionally high specific surface areas and tunable pore structures. They have been widely explored for applications in catalysis,^1–5^ gas adsorption,^6–8^ water treatment,^9–11^ water generation,^12–15^ and biomedical applications.^16–18^ MOFs are typically synthesized as powders via solvothermal or hydrothermal synthesis methods. However, for practical applications in industrial environments,^19–21^ MOFs must be processed into pellets,^22–24^ monoliths,^25–27^ thin films,^28–30^ or membranes.^31–34^ Among these, MOF membranes have shown particular promise for gas separation. Due to their selective transport mechanisms and preferential adsorption behavior, MOF membranes can effectively discriminate between gas molecules. These advantages make them strong candidates for energy-efficient separation processes in industrial gas purification^35–37^ and CO_2_ capture.^38–40^

The earliest studies demonstrating MOF membranes for gas separation were reported in 2009.^41,42^ These seminal works demonstrated the successful synthesis of continuous MOF-5 membranes on porous α-alumina substrates using different fabrication strategies, and both exhibited good hydrogen separation performance. In recent years, Li et al. fabricated UiO-66 membranes via heterogeneous nucleation assisted growth.^43^ The resulting membranes exhibited CO_2_/N_2_ separation performance with a selectivity of 24.3 and a CO_2_ permeance of approximately 530 GPU under 100% relative humidity. Wang et al. fabricated preferentially *c*-oriented MIL-125 membranes with a high density of linker deficiencies via tertiary growth under single-mode microwave irradiation.^44^ The resulting membranes exhibited mixed-gas CO_2_/N_2_ separation performance with a separation factor of 48.3 and a CO_2_ permeance of 127.7 GPU under a 50/50 gas mixture. Wang et al. fabricated MIL-160 tubular membranes using an in situ synthesis method. The resulting membranes exhibited mixed-gas CO_2_/N_2_ separation performance with a separation factor of up to 259 under a 50/50 gas mixture, and CO_2_/CH_4_ separation performance with a separation factor of 224 under a 20/80 gas mixture.^45^

The CAU-series MOFs, developed by the group of Norbert Stock at Christian-Albrechts-University of Kiel,^46–49^ have attracted considerable attention for gas separation. Chuang et al. fabricated dense CAU-23 membranes via thermal and methanol activation for CO_2_ separation. The resulting membranes exhibited CO_2_/N_2_ and CO_2_/CH_4_ separation performance with separation factors of up to 95.3 and 318, respectively, both under a 20/80 gas mixture, with a CO_2_ permeability of approximately 200 Barrer at 3 bar and 35 °C.^50^ Chiou et al. fabricated dense CAU-10-H membranes for CO_2_ separation. The resulting membranes exhibited CO_2_/CH_4_ separation performance with a selectivity of up to 95 and a CO_2_ permeability of around 500 Barrer at 3 bar and 30 °C, attributed to strong Coulombic interactions.^51^ Chang et al. fabricated a CAU-10-PDC-H (70/30) membrane using a mixed-linker strategy. The resulting membrane exhibited mixed-gas CO_2_/CH_4_ separation performance with a separation factor of up to 74.2 under a 50/50 gas mixture at 2 bar and 35 °C.^52^

CAU-1-NH_2_, an Al-based MOF, was first synthesized in 2009.^53^ Its powdered form has also been shown to exhibit potential for applications in wastewater treatment,^54^ ion adsorption,^54^ and gas separation.^55^ The framework exhibits a pore-limiting diameter (PLD) of 4.0 Å (Figure 1). The PLD is comparable to the kinetic diameters of small gas molecules such as H_2_ (2.89 Å), CO_2_ (3.30 Å), N_2_ (3.64 Å), and CH_4_ (3.80 Å), suggesting its potential suitability for membrane-based gas separation. Previous work has shown that CAU-1-NH_2_ membranes exhibit promising CO_2_/N_2_ separation performance in single-gas permeation tests.^56^

**Figure 1:**
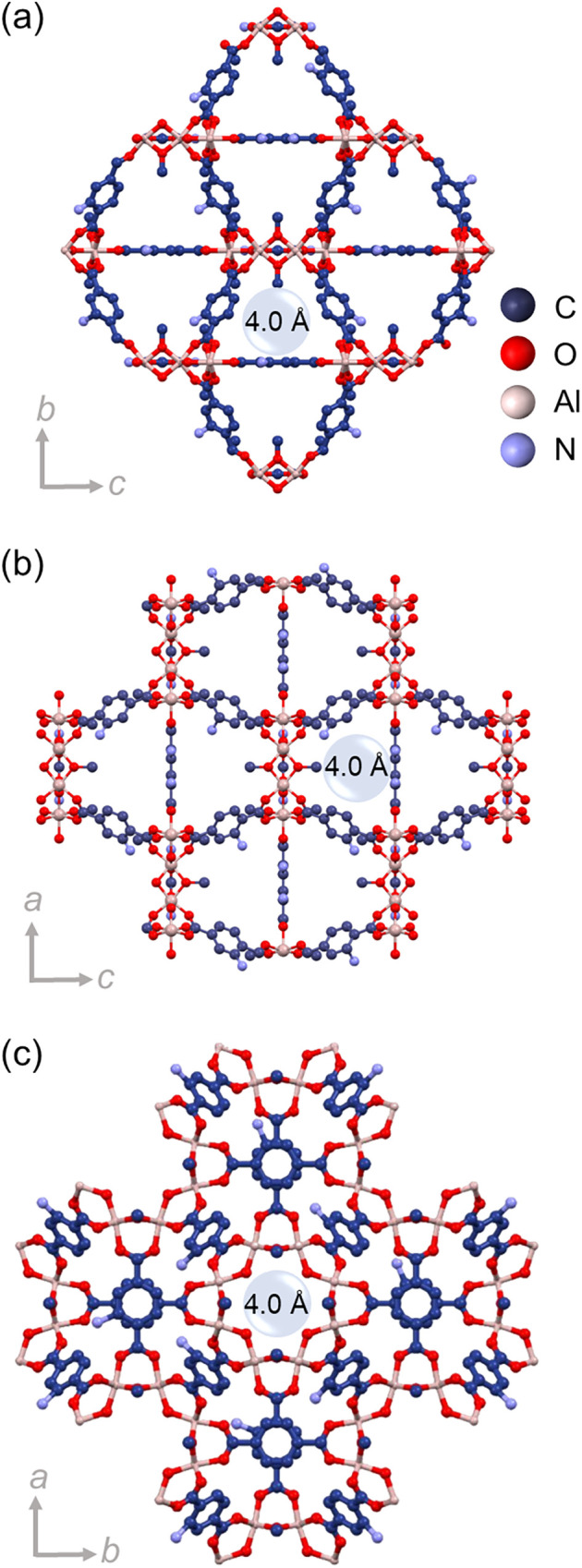
Illustration of the crystal structure and channel widths of CAU-1-NH_2_ viewed along the crystallographic (a) *a*-, (b) *b*-, and (c) *c*-axes.

In this study, we focused on optimizing the fabrication of CAU-1-NH_2_ membranes using a seeded growth approach.^50,52,57^ We systematically examined how key parameters in the secondary growth process—specifically the precursor concentration and the ligand-to-metal molar ratio—affect membrane morphology and gas separation performance. Five CAU-1-NH_2_ membrane variants were synthesized and characterized. Detailed morphological analyses using scanning electron microscopy (SEM) and confocal laser scanning microscopy were performed to evaluate membrane continuity and identify the presence of defects. Single-gas permeation tests with H_2_, CO_2_, N_2_, and CH_4_ were conducted to assess molecular transport behavior and separation characteristics. Additionally, mixed-gas permeation measurements with CO_2_/N_2_ feed mixtures of varying compositions were performed on the optimized membrane to evaluate its applicability for postcombustion CO_2_ capture.

## Experimental Section

### Chemicals and Materials

All chemicals were used as received without any additional purification. Aluminum chloride hexahydrate (99%) and 2-aminoterephthalic acid (NH_2_–H_2_BDC, C_8_H_7_NO_4_, 99%) were obtained from Sigma-Aldrich. Methanol (99%) was sourced from Macron. Deionized water (DI water) was produced using an ELGA VEOLIA PURELAB ultrapure water system. Porous α-alumina disk substrates were procured from YOMO TECHNOLOGY ENTERPRISE Co. These disks had a diameter of 40 mm, a thickness of 2 mm, and a porosity of 34%. The substrates were composed of α-alumina particles with an average particle size of approximately 400 nm.

### Synthesis of CAU-1-NH_2_ Powder

CAU-1-NH_2_ powder was synthesized using a modified procedure based on a previously reported method.^53^ First, aluminum chloride hexahydrate (1.242 g, 5.14 mmol) and 2-aminoterephthalic acid (0.292 g, 1.61 mmol) were dissolved in 50 mL of methanol. The mixture was subjected to ultrasonic agitation for approximately 10 min to ensure complete dissolution and homogeneity. The resulting solution was then transferred to a Teflon-lined stainless-steel autoclave and heated at 125 °C for 5 h under solvothermal conditions. After the reaction, the resulting cloudy yellow suspension was centrifuged at 10,000 rpm for 5 min, followed by washing once with deionized water. The recovered solid was then dried overnight in a vacuum oven at 60 °C. Finally, the dried material was ground into a fine powder and further dried in a conventional oven at 105 °C for at least 24 h prior to use.

### Preparation of CAU-1-NH_2_ Seed Layer

A seed suspension was prepared by dispersing 0.091 g of CAU-1-NH_2_ powder in 9 g of methanol. The suspension was sonicated for 15 min to ensure uniform dispersion of the particles. The resulting suspension was then applied to a porous alumina substrate via spin-on deposition using a Laurell spin coater (Model WS-650MZ-23NPPB) operated at 3000 rpm for 30 s. This deposition step was repeated three times using freshly prepared CAU-1-NH_2_ suspension for each cycle. Following the deposition, the seeded substrate was dried at 105 °C overnight before undergoing secondary growth.

### Secondary Growth of CAU-1-NH_2_ Membranes

The seeded α-alumina substrate was secured in a custom Teflon holder (Figure S1) and placed into a 225 mL Teflon-lined stainless-steel autoclave with the seeded surface facing downward. A secondary growth solution was then introduced, and the reaction was conducted at 125 °C for 7 h, which is a longer reaction time compared to powder synthesis (5 h), to allow sufficient crystal growth and intergrowth on the substrate surface. The standard secondary growth solution consisted of AlCl_3_·6H_2_O (1.553 g, 6.43 mmol), NH_2_–H_2_BDC (0.365 g, 2.02 mmol), and 50 mL of methanol. Upon completion of the reaction, the membrane was thoroughly rinsed with deionized water to remove residual particulates and then dried at 105 °C for 24 h to ensure complete removal of moisture prior to further characterization or application. Under these conditions, the Al^3+^ concentration and ligand-to-metal molar ratio were 0.13 M and 0.33, respectively. To investigate the influence of secondary growth conditions, these two parameters were systematically varied to produce five membrane samples, denoted as CAU-1-NH_2_(A) through CAU-1-NH_2_(E). The detailed compositions of the secondary growth solutions are summarized in Table S1.

### Materials Characterization

In-house X-ray diffraction (XRD) measurements were conducted using a Rigaku SmartLab SE diffractometer equipped with Cu Kα radiation, operated at 40 kV and 40 mA. For both the powder and membrane samples, diffraction patterns were collected over a 2θ range of 5° to 45°, using a step size of 0.02° and a scanning rate of 8° min^–1^.

SEM analysis was conducted using a Hitachi S-4800 field-emission scanning electron microscope (FE-SEM) to examine the surface morphology of both powder and membrane samples. Prior to imaging, samples were sputter-coated with a thin gold layer at a current of 35 mA for 30 s to improve conductivity. The images were captured at an accelerating voltage of 10 kV.

The nitrogen adsorption isotherm at 77 K and the CO_2_, N_2_, and CH_4_ adsorption isotherms at 35 °C were measured using a Micromeritics Tristar II Plus analyzer. A powdered sample was loaded into the analysis tube. Prior to the measurements, the sample was degassed overnight at 150 °C to remove adsorbed water. The pore size distribution was subsequently derived from the N_2_ adsorption isotherm at 77 K using density functional theory (DFT).

Thermogravimetric analysis (TGA) was performed using a TA Instruments SDT 650 system. Approximately 5 mg of the sample was loaded into an alumina crucible. The temperature was increased from room temperature to 800 °C at a heating rate of 10 °C/min. During the measurement, air was introduced at a flow rate of 100 mL/min.

A Leica TCS SP8 X confocal microscope was employed to examine surface defects in the membranes. Membrane samples were immersed in a 1 mM fluorescein sodium solution for 3 days and then allowed to dry overnight in a 105 °C oven prior to imaging. Images were captured using a 405 nm laser along with a white light laser, with emitted light detected within the 497–503 nm range.

### Membrane Gas Permeation

Membrane gas permeation tests were carried out using the constant-volume method with a custom-designed apparatus (Figure S2). For single-gas permeation measurements, the membrane was placed inside a stainless-steel chamber, sealed with aluminum foil tape and an O-ring. To ensure airtight sealing, epoxy (3M DP-100) was applied around the interface between the membrane edge and the foil tape. The assembled membrane cell was placed in a convection oven to control the temperature during both the degassing and permeation processes.

For the permeation test, the feed side of the membrane was exposed to the target gas (H_2_, CO_2_, N_2_, or CH_4_) at an absolute pressure of 3 bar, while the temperature was held at 35 °C. As gas permeated through the MOF membrane, the pressure on the permeate side increased gradually and was continuously monitored using a pressure transducer (MKS AA09A12TCE0). The permeance and permeability of species *i* through the membrane were determined using the following equations:^58^

1 $$Permeance\;of\;species\;i=\frac{V}{R\times T\times A\times \Delta p}\left(\frac{dp_{i}}{dt}\right)$$

2 $$Permeability\;of\;species\;i=\frac{V\times l}{R\times T\times A\times \Delta p}\left(\frac{dp_{i}}{dt}\right)$$

where *l* is the membrane thickness (which can be obtained from SEM images), *A* is the effective membrane area, and *V* is the volume of the permeate side, *Δp*_*i*_ refers to the transmembrane pressure difference of species *i* (which can be assumed to be 3 bar due to the very low downstream pressure), *R* is the gas constant, and *T* is the temperature. $\frac{dp_{i}}{dt}$ refers to the rate of pressure increase on the permeate side.

Binary gas permeation tests were conducted using CO_2_/N_2_ and CO_2_/CH_4_ mixtures with different mole fractions of CO_2_ ( $X_{CO_{2}}$ = 0.05, 0.1, 0.3, or 0.5) at a total absolute pressure of 3 bar and a temperature of 35 °C. The procedure mirrored that of the single-gas permeation tests. The gas composition on the permeate side was analyzed using gas chromatography (GC, Shimadzu GC-2030), with argon as a sweep gas to carry the gases on the permeate side to the detector. The GC was equipped with a thermal conductivity detector and a Shincarbon-ST column to ensure accurate analysis. The separation factor *α*_*i*/_ *_j_* for the binary mixture through the membrane was determined using the following equation:^58^

3 $$\alpha_{i/j}=\frac{y_{i}/y_{j}}{x_{i}/x_{j}}$$

where *x*_*i*_ and *y*_*i*_ represent the molar fractions of component *i* on the feed and permeate sides, respectively, while *x*_*j*_ and *y*_*j*_ represent the corresponding molar fractions for component *j*.

### Molecular Simulations

Single-component and mixed-gas adsorption behaviors of CO_2_, N_2_, and CH_4_ in CAU-1-NH_2_ were simulated using the grand canonical Monte Carlo (GCMC) method implemented in the open-source RASPA^59^ software. The crystal structure of CAU-1-NH_2_ employed in these calculations was obtained from the Cambridge Crystallographic Data Centre (CCDC, deposition number 723320) with some modifications. Specifically, the structure was first converted to P1 symmetry, and fractionally occupied nitrogen atoms on the benzene rings were removed to ensure the presence of exactly one amine group per ligand. Missing hydrogen atoms on the organic ligands were also added. Besides, residual methyl groups on the metal–oxo nodes were removed with the corresponding oxygen atoms saturated with hydrogen for charge neutrality. The CAU-1-NH_2_ framework was treated as rigid in all simulations. To describe intermolecular interactions, nonbonded 12–6 Lennard-Jones (L-J) potential and Coulombic interactions with static point charges were considered. The former was truncated and shifted at a cutoff radius of 12.0 Å, while the latter was calculated using the Ewald summation technique with an accuracy of 10^–6^. The adsorbate molecules (CO_2_, N_2_, and CH_4_) were modeled using the TraPPE force fields.^60,61^ The L-J parameters for the framework atoms were adopted from the DREIDING force field,^62^ while their atomic charges were assigned using the multilayer connectivity-based atom contribution (m-CBAC) method developed by Zou et al.^63^ The Lorentz–Berthelot mixing rule was applied for the L-J parameters between dissimilar atoms. Millions of Monte Carlo attempts, including translation, rotation, reinsertion, and swaps, were carried out to ensure statistically meaningful averages. Partial atomic charges for the framework were assigned using the multilayer connectivity-based atom contribution (m-CBAC) method.

## Results and Discussion

### Characterization of CAU-1-NH_2_ Powder

We first characterized the intrinsic properties of CAU-1-NH_2_ powder, as these can influence membrane gas separation performance. The powder XRD pattern of the as-synthesized CAU-1-NH_2_ is shown in Figure S3, alongside the simulated pattern for comparison. Overall, the experimental pattern closely matched the simulated one, with a few minor deviations. Notably, the relative intensity of the (101) peak in the experimental pattern was slightly lower than that in the simulated pattern, which may suggest the presence of preferred orientation in the powder sample. Additionally, the (200) and (002) peaks appeared less resolved in the experimental pattern than in the simulated one. This peak merging may be attributed to factors such as peak broadening caused by small crystallite size. Internal strain within the crystals, or slight deviations in crystallinity resulting from the synthesis conditions.

The morphology of the CAU-1-NH_2_ powder was further examined by SEM, as shown in Figure S4. The images reveal near-spherical particles with a uniform size of approximately 50 nm. Such a small crystallite size is expected to result in significant peak broadening in XRD, which likely accounts for the merging of the (200) and (002) reflections observed in the experimental XRD pattern of the as-synthesized CAU-1-NH_2_ powder.

The TGA results of CAU-1-NH_2_ powder are shown in Figure S5. A weight loss of approximately 25% was observed below 85 °C, likely due to the desorption of physically adsorbed water. The sharp mass loss at such a low temperature suggests that water is only weakly bound to the framework, indicating minimal interaction between water molecules and the CAU-1-NH_2_ structure. The crystalline framework remained stable up to approximately 300 °C, beyond which structural decomposition occurred. These findings are consistent with previous reports in the literature.

To evaluate the porosity of CAU-1-NH_2_, nitrogen adsorption measurements were conducted at 77 K (Figure 2). The isotherm exhibited characteristics typical of a microporous material. A noticeable increase in nitrogen uptake at high relative pressures (*P*/*P*_0_ > 0.9) was also observed, which may be attributed to the presence of small particles with significant external surface area. Pore size distribution analysis based on the adsorption isotherm revealed a dominant pore size of approximately 9 Å, which is in good agreement with the pore features predicted from the crystallographic structure deposited in CCDC (No. 723320) using the open-source software Zeo++, yielding a predicted pore size of approximately 10.5 Å.^64^ This observation represents that the experimentally accessible pore volume is well correlated with the ideal crystal structure. To evaluate gas solubility in CAU-1-NH_2_, adsorption isotherms of CO_2_, N_2_, and CH_4_ were measured at 35 °C, as shown in Figure S6. Among the three gases, CO_2_ exhibited the highest adsorption uptake, followed by CH_4_ and N_2_. At 1 bar, the CO_2_ uptake reached 2.93 mmol/g, consistent with previously reported values. The calculated adsorption selectivities for CO_2_/N_2_ and CO_2_/CH_4_ at 1 bar were approximately 17.2 and 4.6, respectively, indicating a strong affinity of CAU-1-NH_2_ for CO_2_ over the other gases. These results highlight the potential of CAU-1-NH_2_ for CO_2_ separation.

**Figure 2:**
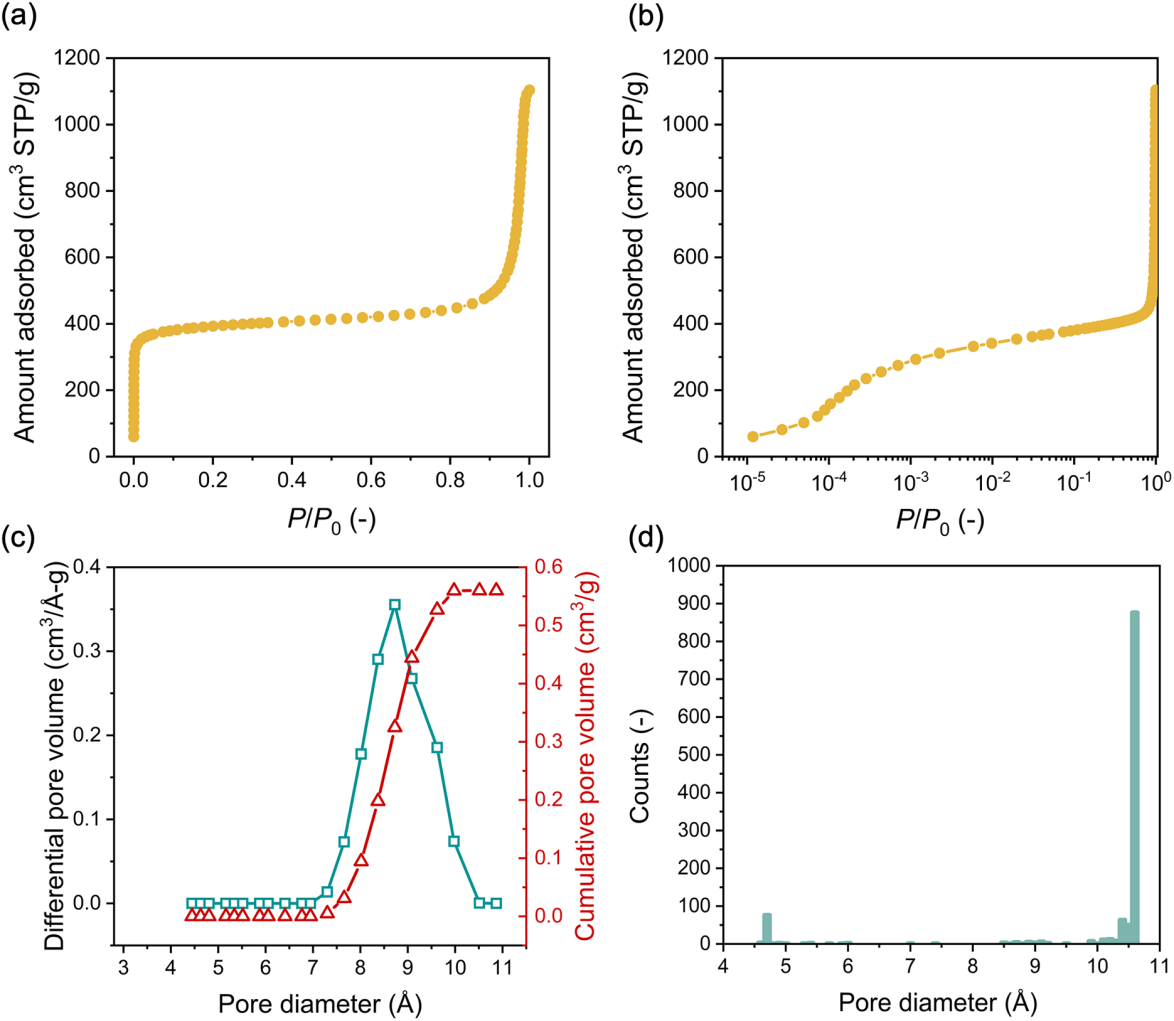
N_2_ adsorption isotherm of CAU-1-NH_2_ powder measured at 77 K, shown on (a) linear and (b) logarithmic scales. (c) Pore size distribution derived from the adsorption branch of the N_2_ isotherm using a DFT model. (d) Pore size distribution calculated from the crystal structure (CCDC 723320) using Zeo++.

To further elucidate the gas adsorption behavior, GCMC simulations were performed for CO_2_, N_2_, and CH_4_ in the pressure range of 1 to 3 bar (Figure S7). Specifically, at 3 bar, the simulated uptakes of CO_2_, CH_4_, and N_2_ reached 8.51, 2.56, and 0.89 mmol/g, respectively. Furthermore, the GCMC-derived adsorption selectivities for CO_2_/N_2_ and CO_2_/CH_4_ under these conditions were calculated to be approximately 9.56 and 3.32, respectively, further corroborating the strong preferential binding of CO_2_ within the CAU-1-NH_2_ framework. These simulation results were then used to calculate the single-gas solubility. The observed discrepancy between simulation and experiment is within the expected range, as simulations assume an ideal, defect-free framework with fully accessible pores, whereas real samples may contain defects, residual solvent, partial pore blocking, or incomplete activation, all of which reduce the accessible adsorption volume.

### Characterization of CAU-1-NH_2_ Membranes

The seeded growth method was employed for the fabrication of CAU-1-NH_2_ membranes. Five CAU-1-NH_2_ membranes were prepared using different secondary growth recipes and are denoted as CAU-1-NH_2_(A) through CAU-1-NH_2_(E). All membranes were fabricated from identical seed layers, while variations were introduced only during the secondary growth step. The compositions of the five secondary growth recipes are summarized in Table S1. SEM images of the bare alumina substrate and the seeded substrate are shown in Figure S8. The porous substrate consists of α-alumina particles with sizes ranging from approximately 200 to 500 nm. After the seeding process, the substrate surface is homogeneously covered with CAU-1-NH_2_ nanoparticles, which serve as nucleation sites for subsequent secondary growth. The dense and uniform distribution of CAU-1-NH_2_ seeds is favorable for promoting low-defect membrane growth.

The SEM images of the five CAU-1-NH_2_ membranes are presented in Figure 3 and Figures S9–S13. In the cross-sectional SEM images, the MOF layer of the CAU-1-NH_2_(A) membrane is barely discernible, in contrast to the other four membrane samples. This is likely due to the relatively low concentration of Al^3+^ used in the secondary growth solution, which likely resulted in a reduced crystal growth rate during membrane formation. Well-defined MOF grains are clearly observed in the top-view SEM images of CAU-1-NH_2_(B) and CAU-1-NH_2_(C), with a similar average grain size of approximately 0.5 μm. In contrast, the crystal morphologies of CAU-1-NH_2_(D) and CAU-1-NH_2_(E) are poorly resolved in the top-view SEM images, despite the presence of a continuous MOF layer evident from the cross-sectional views. This behavior can be attributed to the relatively higher ligand-to-metal ratio which is employed during the secondary growth of CAU-1-NH_2_(D) and CAU-1-NH_2_(E), leading to suppressed nucleation and a less distinct surface morphology.

**Figure 3:**
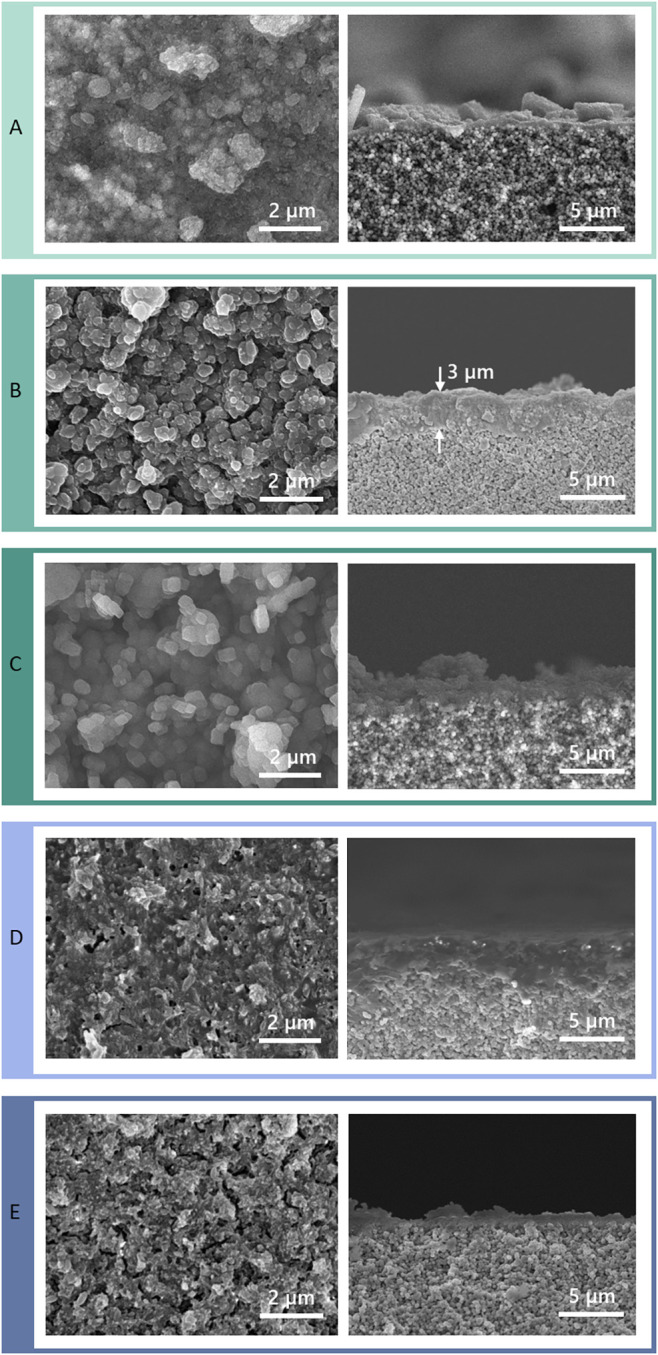
Top-view (left) and cross-sectional (right) SEM images of CAU-1-NH_2_ membranes (A–E), arranged from top to bottom.

The XRD patterns of the five CAU-1-NH_2_ membrane samples, shown in Figure 4, further support the morphological observations from the SEM analysis. Specifically, CAU-1-NH_2_(B) and CAU-1-NH_2_(C) exhibit higher signal-to-noise ratios than the other three samples, indicating better-developed MOF crystal structures in these two membranes. Moreover, these two membranes still exhibit a dominant (101) reflection, indicating that the membranes remain largely polycrystalline. Nevertheless, the gradual increase in the relative intensity of the (002) peak suggests the emergence of a weak preferred orientation, which may be associated with the partial alignment of the *c*-axis perpendicular to the substrate surface. A more pronounced change in crystal orientation is observed for the CAU-1-NH_2_(D) and CAU-1-NH_2_(E) membranes. In these samples, the intensity of the (101) reflection decreases substantially, while the (211) reflection becomes dominant, indicating the development of a preferred membrane orientation promoted by the higher ligand-to-metal ratio employed during secondary growth, which favors the formation of (211)-oriented crystal domains. The CAU-1-NH_2_(A) membrane exhibits very weak diffraction signals for all reflections. Consistent with the SEM observations, this is likely due to its poor MOF surface coverage.

**Figure 4:**
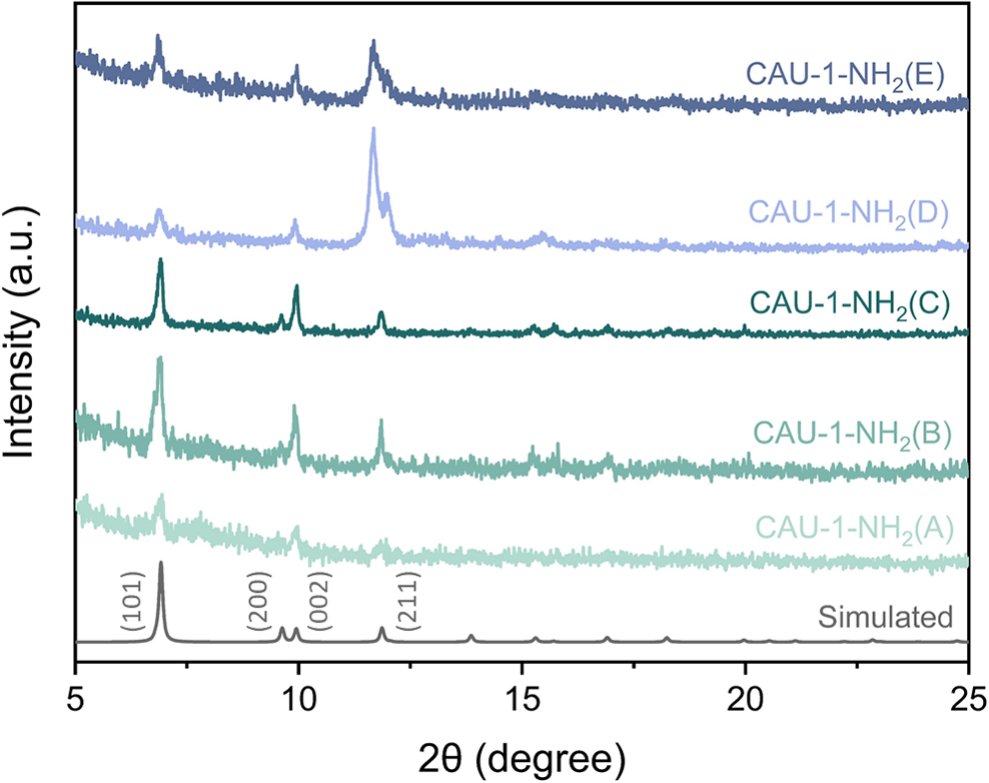
XRD patterns of the five CAU-1-NH_2_ membranes. The simulated powder XRD pattern of CAU-1-NH_2_ is included for comparison.

Confocal fluorescence microscopy images of the five CAU-1-NH_2_ membranes stained with fluorescein sodium are shown in Figure 5. This technique is a well-established method for identifying pinhole-type defects in polycrystalline zeolite^65,66^ and MOF^45,50^ membranes. Fluorescein sodium was introduced to infiltrate grain-boundary voids and defect sites, which were subsequently visualized by confocal fluorescence microscopy. The white lines indicate the corresponding cross-sectional regions, while the circles highlight the locations of detected defects. With the exception of CAU-1-NH_2_(B), all CAU-1-NH_2_ membrane samples exhibit pronounced fluorescence spots, indicating localized accumulation of fluorescein sodium. These observations suggest the presence of pinhole-type defects in the CAU-1-NH_2_(A), CAU-1-NH_2_(C), CAU-1-NH_2_(D), and CAU-1-NH_2_(E) membranes. The fluorescence area percentages of the five samples were quantified using ImageJ image analysis, and the corresponding area fractions were 23.4%, 98.8%, 85.8%, 83.9%, and 17.2% for CAU-1-NH_2_(A)–(E), respectively. These results confirm that CAU-1-NH_2_(B) exhibits the highest surface coverage among all samples.

**Figure 5:**
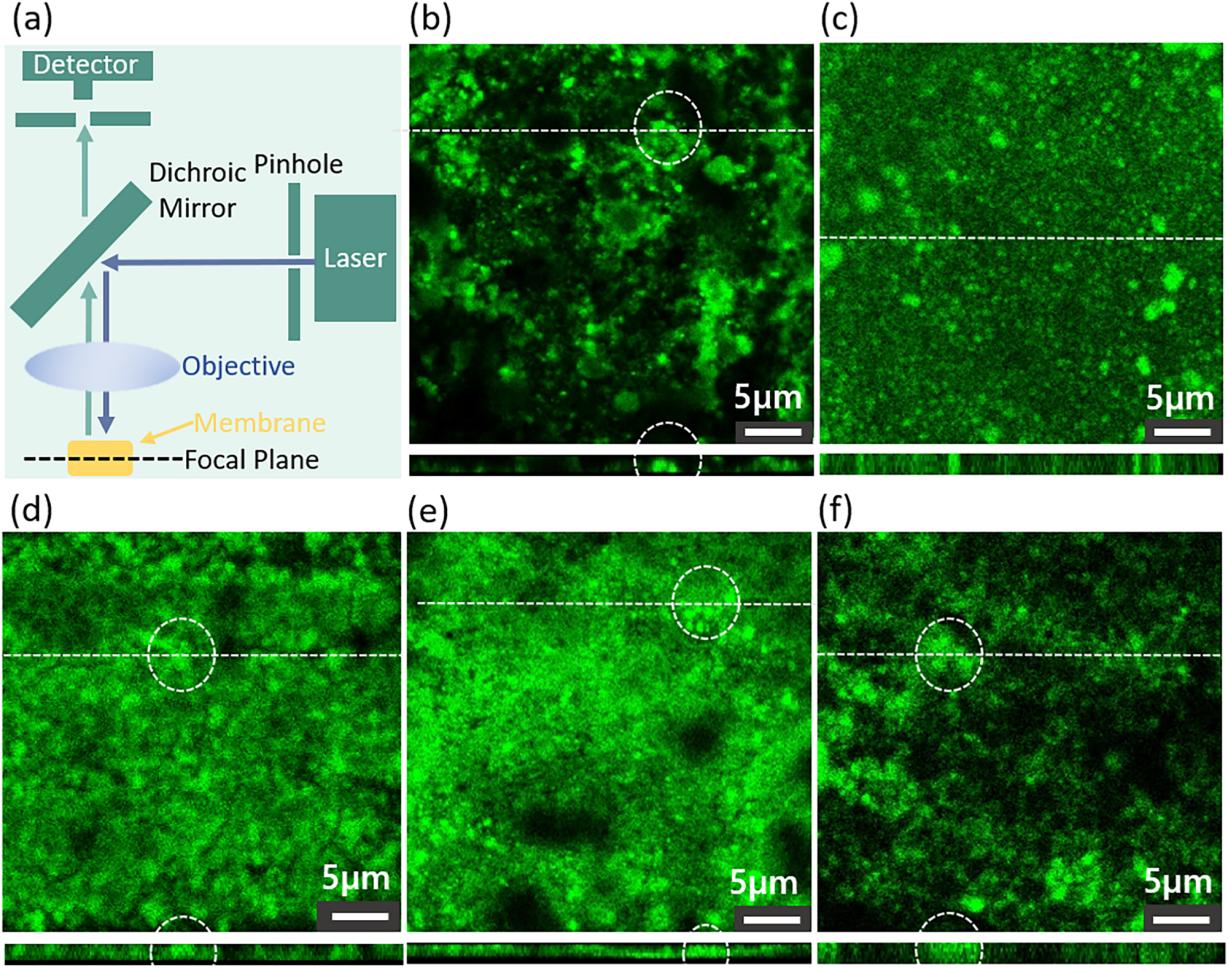
(a) Schematic illustration of the confocal fluorescence microscopy setup. (b–f) Confocal fluorescence microscopy images of CAU-1-NH_2_ membranes (A–E), showing top-view (upper) and cross-sectional (lower) images for each membrane. Fluorescein sodium staining was used to visualize grain-boundary voids and pinhole-type defects. White lines indicate the locations of the corresponding cross-sectional views, and circles highlight identified defects.

Single-gas permeation measurements of H_2_, CO_2_, N_2_, and CH_4_ were conducted at 35 °C under a feed pressure of 3 bar to evaluate the gas transport properties of the membranes. The corresponding gas permeances, together with the ideal CO_2_/N_2_ and CO_2_/CH_4_ selectivities (calculated as the ratios of single-gas permeances), are summarized in Figure 6. For comparison, the theoretical selectivities based on Knudsen diffusion are also presented in Figure 6b. Under the Knudsen diffusion model, gas diffusivities are inversely proportional to the square root of the molecular weight of the permeating species. Knudsen diffusion typically occurs in grain boundary voids,^67,68^ which are significantly larger than the intrinsic micropores of MOFs. Therefore, when the experimentally measured selectivities approach the Knudsen values, it suggests that gas permeation is governed by mesoporous pinholes or grain-boundary defects, rather than by molecular sieving through the intrinsic channels of the MOF framework.^45,50^

**Figure 6:**
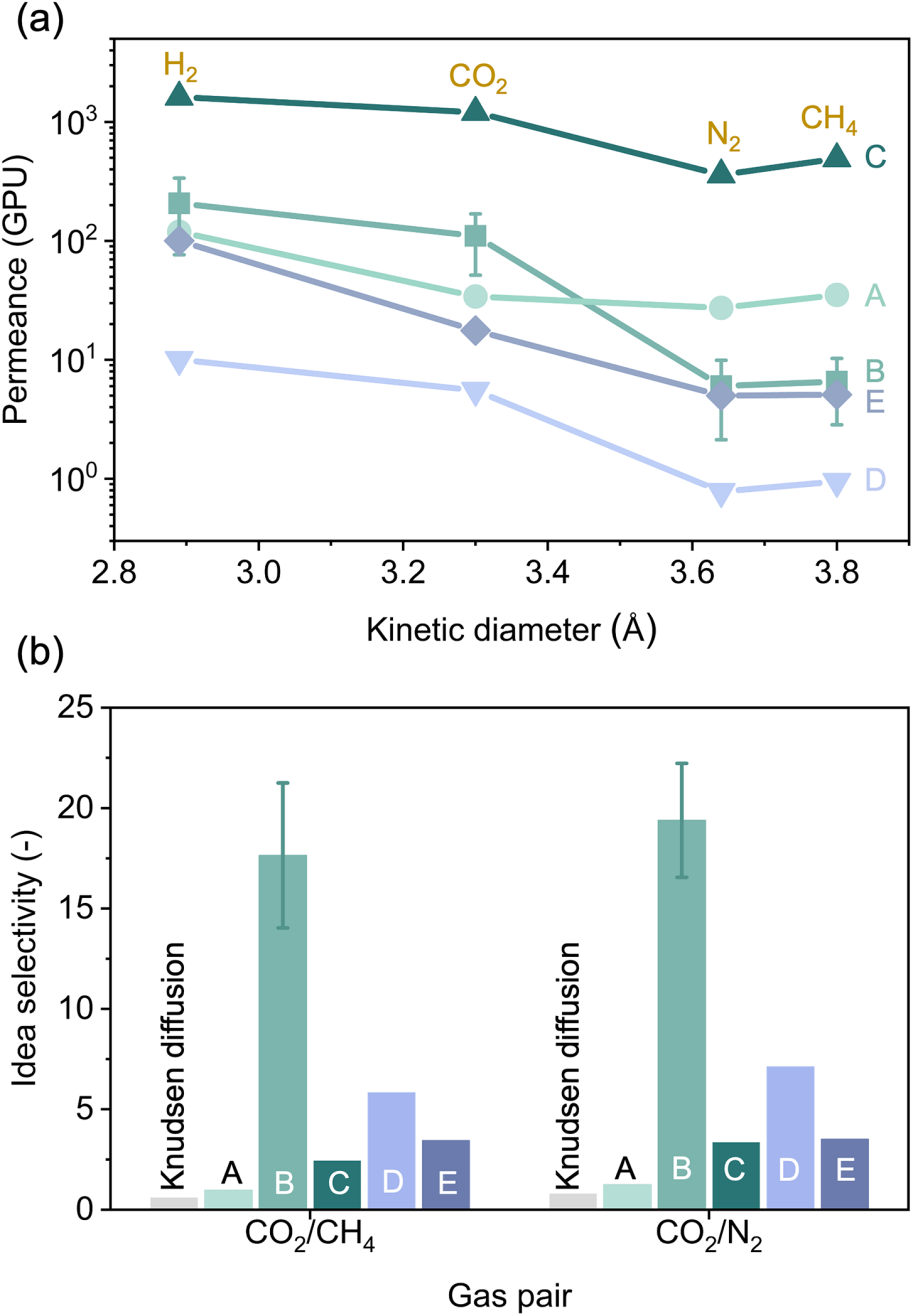
(a) Single-gas permeances of H_2_, CO_2_, N_2_, and CH_4_ through the CAU-1-NH_2_ membranes measured at 35 °C and a feed pressure of 3 bar; (b) corresponding ideal CO_2_/CH_4_ and CO_2_/N_2_ selectivities.

Among these membranes, CAU-1-NH_2_(A) exhibits low crystallinity, as suggested by XRD analysis. However, the presence of pinhole-like defects leads to poor selectivity. CAU-1-NH_2_(C) exhibits significantly higher gas permeance than the other four samples. This behavior may be attributed to its better-developed crystal structure, as suggested by XRD analysis. However, it also generates macroscopic grain-boundary defects. These defects act as nonselective pathways for gas molecules, resulting in extremely high gas permeance but severely compromising selectivity. The XRD results also show that CAU-1-NH_2_(B) possesses a crystallinity comparable to that of CAU-1-NH_2_(C). The CAU-1-NH_2_(B) membrane exhibits the second-highest H_2_ and CO_2_ permeances among the five samples, only slightly lower than those of CAU-1-NH_2_(C), while displaying substantially higher CO_2_/N_2_ and CO_2_/CH_4_ selectivities. This observation suggests that fewer pinhole defects are present in the CAU-1-NH_2_(B) membrane compared with CAU-1-NH_2_(C).

When the ligand-to-metal ratio was varied, particularly at higher ligand-to-metal ratios, nucleation was suppressed, leading to a reduced nucleation rate, incomplete reaction, and consequently the formation of defects and a poorly developed surface morphology. As a result, CAU-1-NH_2_(D) exhibited a significantly thicker membrane layer and lacked distinct surface grain boundaries. However, both CAU-1-NH_2_(D) and CAU-1-NH_2_(E) retained internal defects, while excess ligands remained trapped within their pores and defect sites. This ligand-induced pore blockage, together with the greater membrane thickness of CAU-1-NH_2_(D), severely restricted gas transport. Consequently, both CAU-1-NH_2_(D) and CAU-1-NH_2_(E) exhibited very low gas permeance and selectivities predicted by Knudsen diffusion.

CAU-1-NH_2_(B) demonstrates the best overall gas separation performance. The H_2_, CO_2_, N_2_, and CH_4_ permeances of the CAU-1-NH_2_(B) membrane are 207.1, 110.2, 6.0, and 6.6 GPU, respectively, corresponding to ideal CO_2_/N_2_ and CO_2_/CH_4_ selectivities of 19.4 and 17.6. Given its favorable combination of relatively high permeance and enhanced selectivity, CAU-1-NH_2_(B) is identified as the optimal sample. Therefore, the subsequent discussion of gas separation performance focuses primarily on this membrane. Notably, despite N_2_ having a smaller kinetic diameter, the CAU-1-NH_2_ membrane consistently exhibited a higher permeance for CH_4_. This behavior can be attributed to the stronger adsorption affinity of CH_4_ within the CAU-1-NH_2_ framework, which compensates for its lower diffusivity. The result highlights the important role of adsorption in governing gas transport through the membrane.

### Gas Separation Performance of CAU-1-NH_2_ Membranes

To further evaluate the gas separation performance of CAU-1-NH_2_, the CAU-1-NH_2_(B) membrane was subjected to binary gas permeation measurements. Specifically, CO_2_/N_2_ and CO_2_/CH_4_ mixtures with varying CO_2_ mole fractions ( $X_{CO_{2}}$ = 0.05, 0.1, 0.3, and 0.5) were tested at a total pressure of 3 bar and a temperature of 35 °C (Figure 7a and b). For the CO_2_/CH_4_ mixtures, the CO_2_ permeance shows only slight variation, ranging from 46.0 to 54.6 GPU across the tested compositions. For the CO_2_/N_2_ mixtures, the CO_2_ permeance ranges from 39.0 to 88.9 GPU. Despite these variations, the measured CO_2_ permeances show a large deviation from those obtained from single-gas measurements (110.2 GPU). These results indicate that CO_2_ transport through the CAU-1-NH_2_(B) membrane is influenced by the presence of another diffusing species (N_2_ or CH_4_).

**Figure 7:**
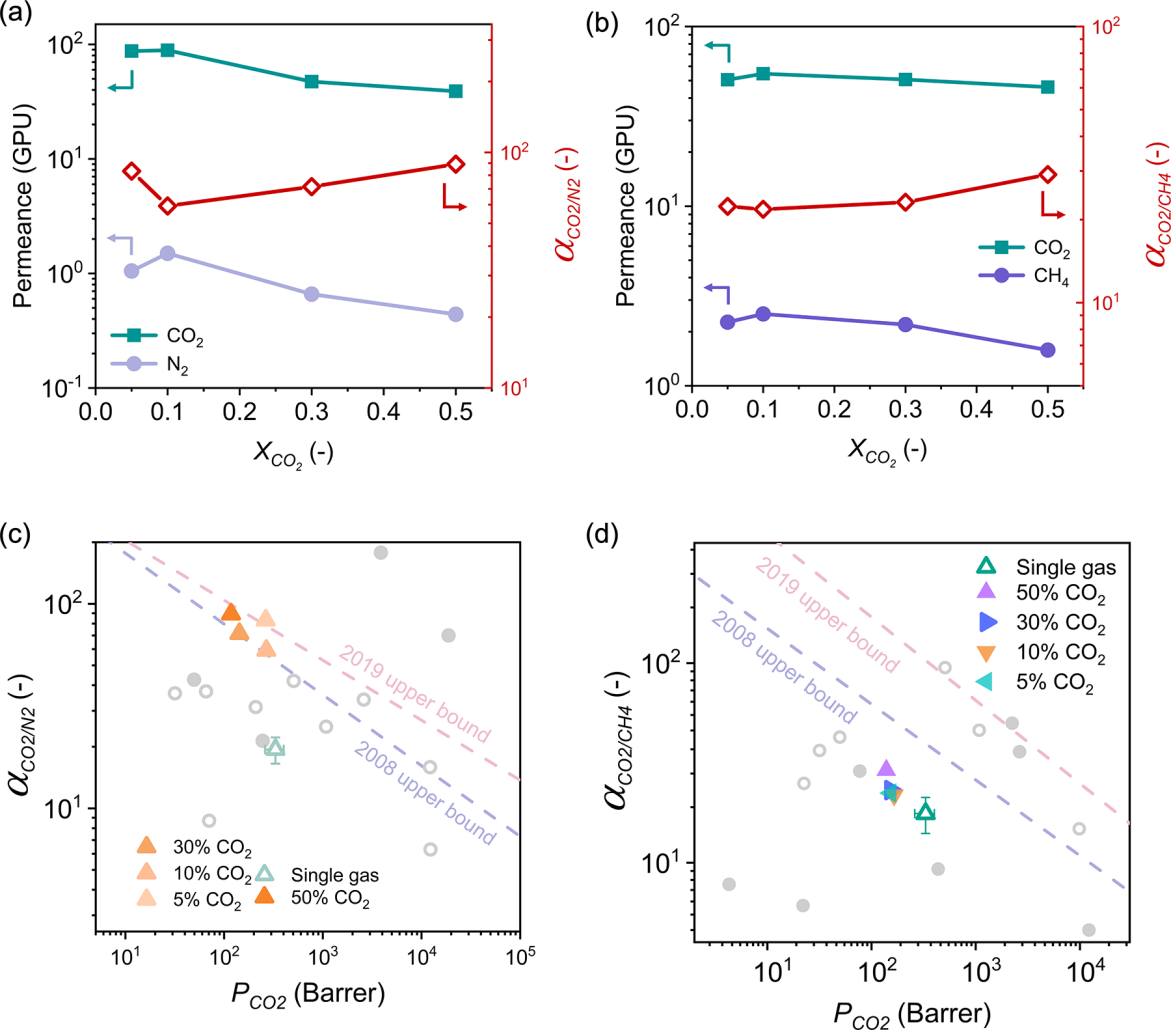
Gas permeance and separation factors of the CAU-1-NH_2_(B) membrane evaluated at 35 °C and a total feed pressure of 3 bar with binary mixtures of (a) CO_2_/N_2_ and (b) CO_2_/CH_4_ at various feed compositions. Robeson-type plots of (c) CO_2_/N_2_ and (d) CO_2_/CH_4_ separation performance of the CAU-1-NH_2_(B) membrane (triangles) compared with previously reported MOF membranes (gray circles). In (c) and (d), open symbols represent results from single-gas permeation measurements, while solid symbols correspond to mixed-gas measurements.

The permeances of N_2_ and CH_4_ in the binary mixtures are significantly lower than those measured under single-gas conditions, indicating that the presence of CO_2_ strongly influences their adsorption and diffusion behavior within the membrane. This behavior can be attributed to the preferential adsorption of CO_2_ in the CAU-1-NH_2_ framework, which suppresses the transport of the less strongly adsorbing gases. As a result, the CAU-1-NH_2_(B) membrane exhibits exceptionally high CO_2_/N_2_ separation factors in the binary gas measurements, ranging from 59.3 to 89.2, which are substantially higher than the corresponding ideal selectivity (19.4). Similarly, enhanced separation performance is observed for the CO_2_/CH_4_ mixtures, although the improvement relative to the single-gas selectivity is less pronounced than that observed for CO_2_/N_2_ separation.^69^

To benchmark the gas separation performance of CAU-1-NH_2_, the separation performance of the CAU-1-NH_2_(B) membrane under various feed compositions is compared with previously reported MOF membranes.^45,51,52,69–78^ The results for CO_2_/N_2_ and CO_2_/CH_4_ separations are shown in Figure 7c and d, respectively. The data presented in these figures are tabulated in Tables S2 and S3. For this comparison, the thickness of the CAU-1-NH_2_(B) membrane was assumed to be 3 μm for converting gas permeances to gas permeabilities. For CO_2_/N_2_ separation, the CAU-1-NH_2_(B) membrane exhibits good separation performance compared with reported MOF membranes. Notably, its overall separation performance exceeds the 2008 polymer upper bound^79^ and one of the results exceeds the 2019 upper bound.^80^ In contrast, the CO_2_/CH_4_ separation performance of the CAU-1-NH_2_(B) membrane is comparatively moderate. Although its performance approaches the 2008 polymer upper bound, it does not exceed it.

To further investigate the origin of the high separation factor of CAU-1-NH_2_ for CO_2_/N_2_ and CO_2_/CH_4_ mixtures, binary gas adsorption in this MOF was simulated using GCMC methods. For a standardized and practical analysis, the simulated adsorption capacities were converted into solubility values to determine the adsorption selectivity (Figure 8a and 8c). The results demonstrate that CO_2_ solubility increases as its mole fraction (X_CO2_ = 0.05, 0.1, 0.3, and 0.5) decreases. Notably, even at lower CO_2_ partial pressure, the CAU-1-NH_2_ framework preferentially adsorbs CO_2_ due to strong framework–adsorbate interactions. As a result, the overall adsorption selectivities for CO_2_/N_2_ and CO_2_/CH_4_ increase with decreasing CO_2_ mole fraction, reaching values of 24.8–46.4 and 6.0–13.5, respectively.

**Figure 8:**
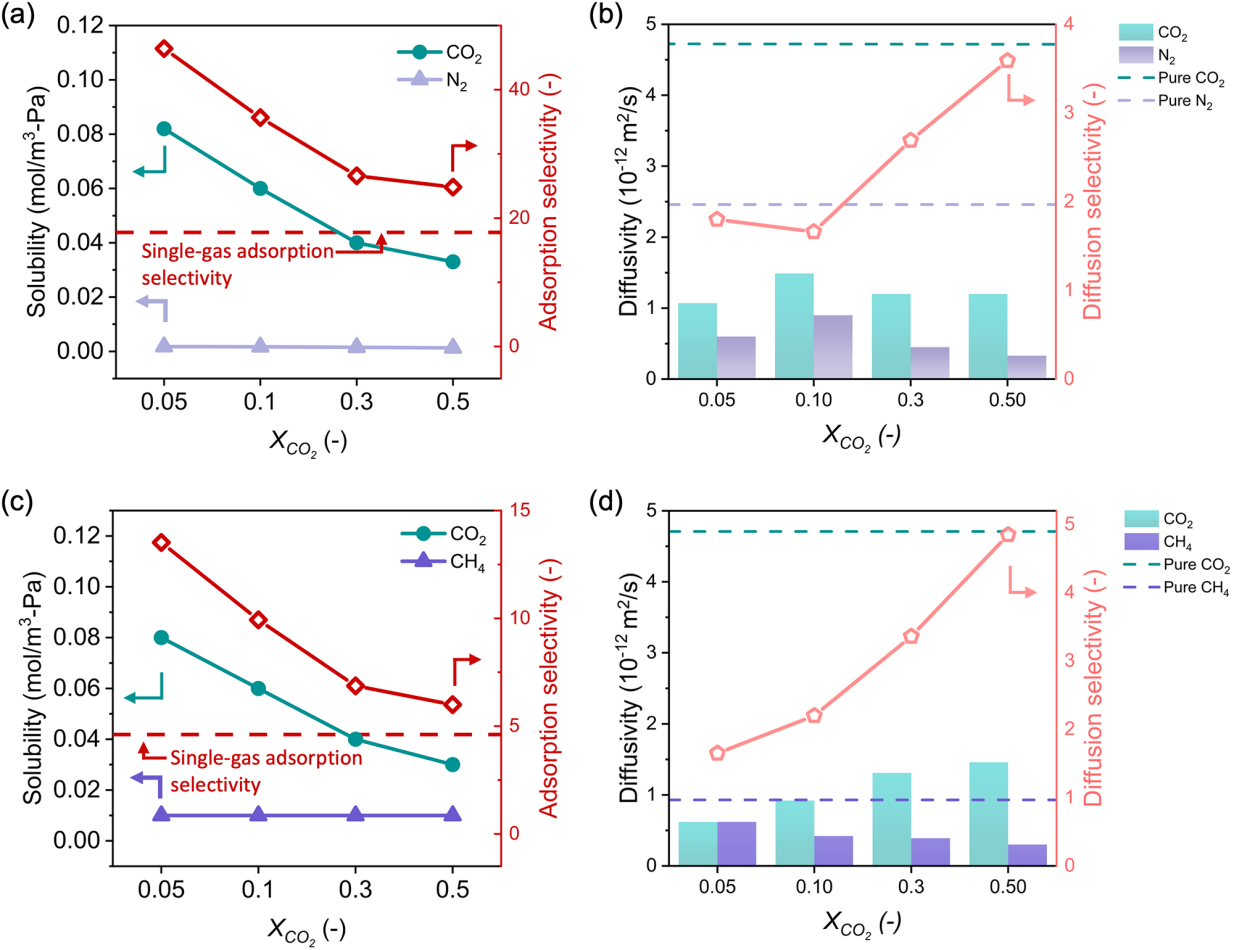
Solubility and adsorption selectivities of CAU-1-NH_2_(B) for (a) CO_2_/N_2_ and (c) CO_2_/CH_4_ mixtures simulated by GCMC as a function of CO_2_ molar fraction at 35 °C. The red dashed lines denote the adsorption selectivities for single gases at 1 bar. Diffusivity and diffusion selectivities of CAU-1-NH_2_(B) for (b) CO_2_/N_2_ and (d) CO_2_/CH_4_ mixtures calculated by the solution-diffusion model as a function of CO_2_ molar fraction. The green and purple lines represent the single-gas diffusivity of CO_2_ and N_2_, respectively.

To elucidate the mixed-gas transport mechanism, single- and mixed-gas diffusivities were estimated using the solution–diffusion model by integrating experimental permeabilities with simulated adsorption data converted into solubility values (Figure 8b and 8d). Under CO_2_/N_2_ and CO_2_/CH_4_ conditions, the diffusivities of both gases decreased compared with their single-component counterparts. This indicates that when different gas molecules are present together in confined pores, they interfere with each other, leading to reduced diffusivity. Consequently, the diffusion selectivities for CO_2_/N_2_ (1.7–3.59) and CO_2_/CH_4_ (1.7–4.9) were significantly lower than the corresponding adsorption selectivities obtained from GCMC simulations of the mixed-gas systems (24.8–46.4 for CO_2_/N_2_ and 6.0–13.5 for CO_2_/CH_4_), indicating that the overall separation performance is predominantly governed by competitive CO_2_ adsorption.

Finally, the long-term stability of the membrane was systematically evaluated to assess its operational reliability for CO_2_/N_2_ separation (Figure 9). In the stability test, the membrane was first evaluated at time zero and then stored under vacuum for 72 h before remeasurement. Both CO_2_ and N_2_ permeances decreased from 61.7 to 39.5 GPU and from 2.7 to 1.8 GPU, respectively, while the selectivity remained nearly unchanged. Subsequently, measurements were conducted every 24 h, during which both permeances gradually decreased, while the selectivity remained nearly unchanged. After 168 h, the permeances further decreased to 33.4 GPU and 1.6 GPU for CO_2_ and N_2_, respectively,

**Figure 9:**
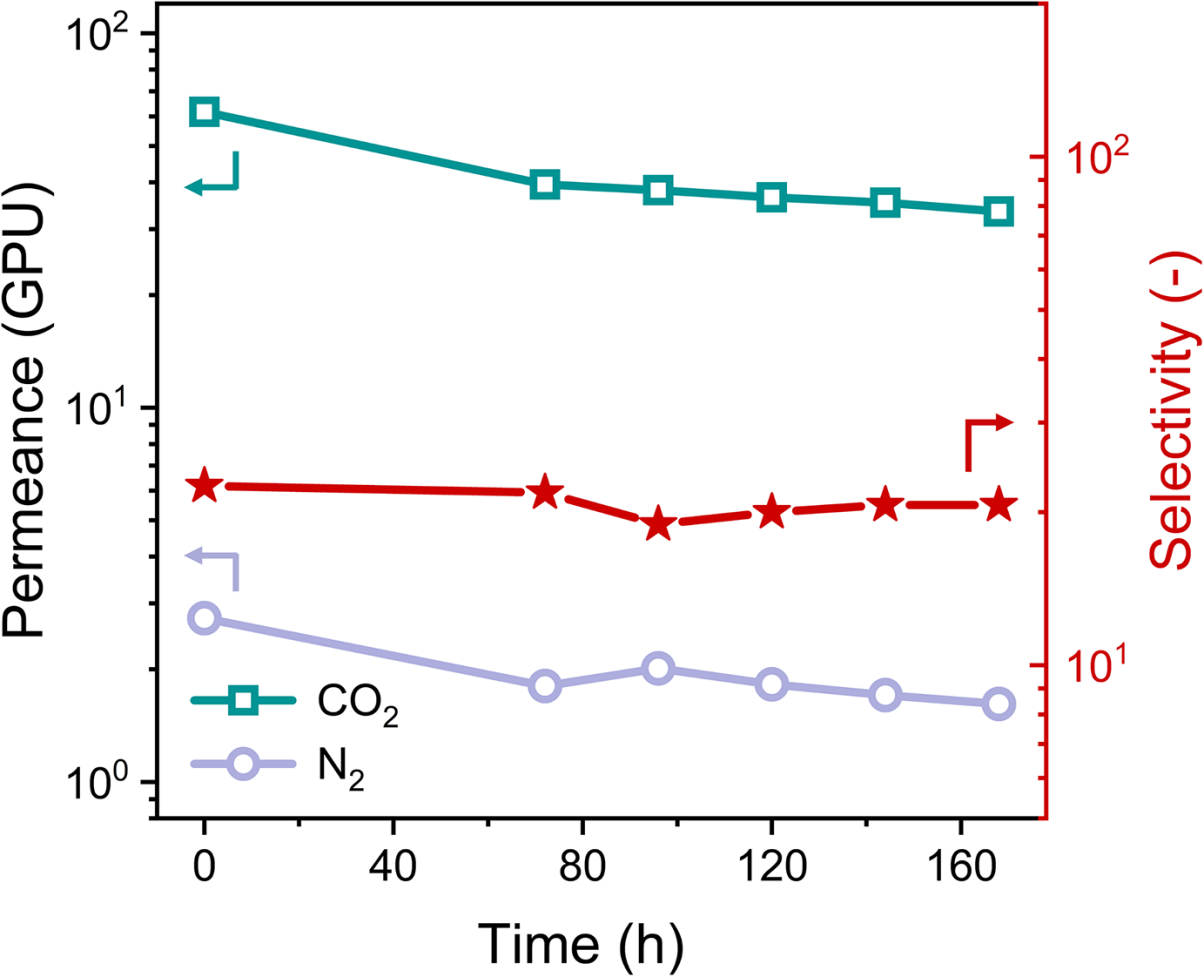
Time-dependent stability test of the CAU-1-NH_2_(B) membrane under single-gas (CO_2_ and N_2_) permeation at 35 °C and 3 bar.

## Conclusions

In this study, CAU-1-NH_2_ membranes were successfully fabricated on porous α-alumina substrates via a seeded growth approach. By systematically varying the precursor concentration and the ligand-to-metal molar ratio, five membrane variants were prepared and evaluated. Among them, the CAU-1-NH_2_(B) membrane exhibited the most favorable combination of crystallinity, membrane continuity, and defect suppression, as confirmed by SEM, XRD, and confocal fluorescence microscopy. The optimized CAU-1-NH_2_(B) membrane achieved H_2_, CO_2_, N_2_, and CH_4_ permeances of 207.1, 110.2, 6.0, and 6.6 GPU, respectively, corresponding to ideal CO_2_/N_2_ and CO_2_/CH_4_ selectivities of 19.4 and 17.6. Mixed-gas permeation measurements revealed substantially enhanced CO_2_ separation performance, with CO_2_/N_2_ separation factors ranging from 59.3 to 89.2. Benchmark analysis showed that the membrane outperformed most reported MOF membranes for CO_2_/N_2_ separation and exceeded the polymeric upper bounds. GCMC simulations further revealed strong competitive adsorption of CO_2_ over N_2_ and CH_4_, indicating that adsorption selectivity plays a dominant role in the observed mixed-gas separation behavior. Overall, this work demonstrates that careful control of growth conditions is critical for producing low-defect CAU-1-NH_2_ membranes with high CO_2_ separation performance. Future studies should focus on evaluating membrane stability under humid and industrially relevant operating conditions to assess the potential of CAU-1-NH_2_ membranes for postcombustion CO_2_ capture from flue gas streams

## Supplementary Material



## Notes

Photographic images of the Teflon holder and a schematic illustration of the membrane permeation setup; XRD patterns, SEM images, TGA results, and gas adsorption isotherms of CAU-1-NH_2_ powder; simulated gas adsorption isotherms from GCMC calculations; SEM images of the seeded substrate and individual CAU-1-NH_2_ membranes; experimental parameters for membrane synthesis; benchmarking data comparing the CO_2_/N_2_ and CO_2_/CH_4_ separation performance of CAU-1-NH_2_ membranes with previously reported MOF membranes (PDF)
