# Supplementary material for: Controlled Secondary Growth of CAU-1-NH2 Membranes with Improved CO2 Separation Performance
Source: Langmuir. 2026 Jul 3;42(28):20537–50. doi: 10.1021/acs.langmuir.6c02353 (PMC13394402; doi:10.1021/acs.langmuir.6c02353)
Supplement: Supplementary file 1 [file la6c02353_si_001.pdf]

Supporting Information

for

Controlled Secondary Growth of CAU-1-NH_2 Membranes with Improved CO_2 Separation Performance

Bing-Han Lin, Hsiang-Yu Wang, Li-Tang Chi, Chia-Hui Chuang, Yi-Hsuan Lin, Li-Chiang Lin,* and Dun-Yen Kang*

Department of Chemical Engineering, National Taiwan University, No. 1, Sec. 4, Roosevelt Road, Taipei 10617, Taiwan

Li-Chiang Lin *E-mail: lclin@ntu.edu.tw

Dun-Yen Kang *E-mail: dunyen@ntu.edu.tw

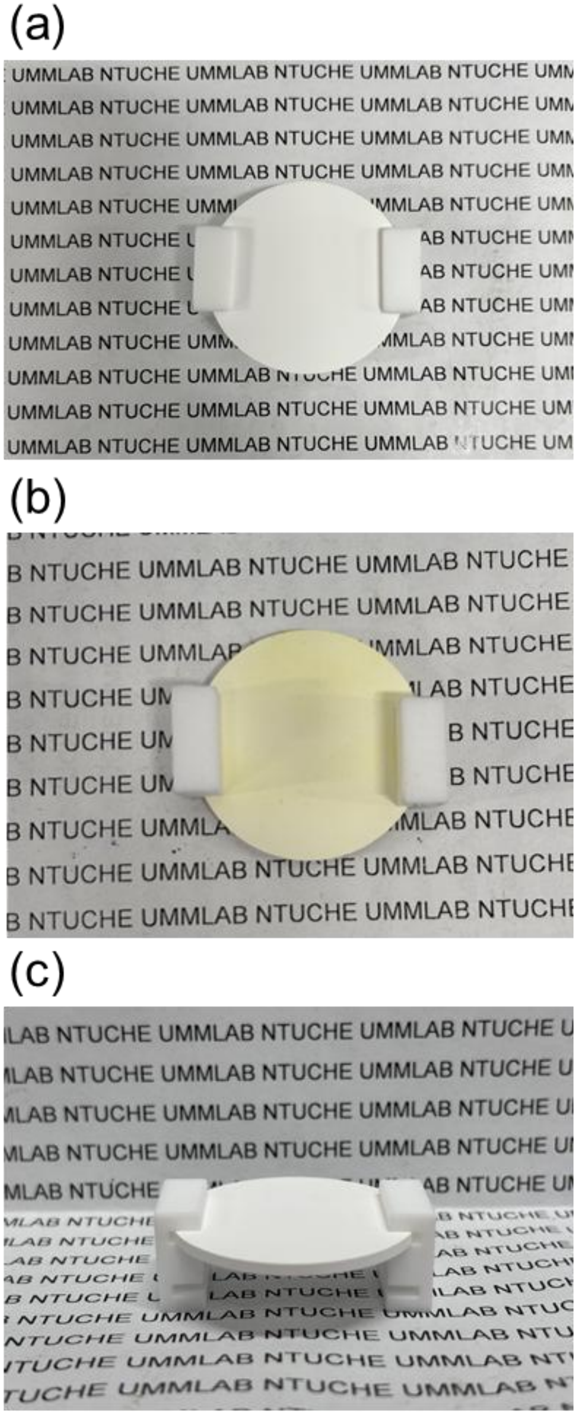

Figure S1. Photographic images of the Teflon holder for the alumina substrate: (a) top view, (b) bottom view showing the CAU-1-NH_2 seed layer grown on the substrate, and (c) side view.

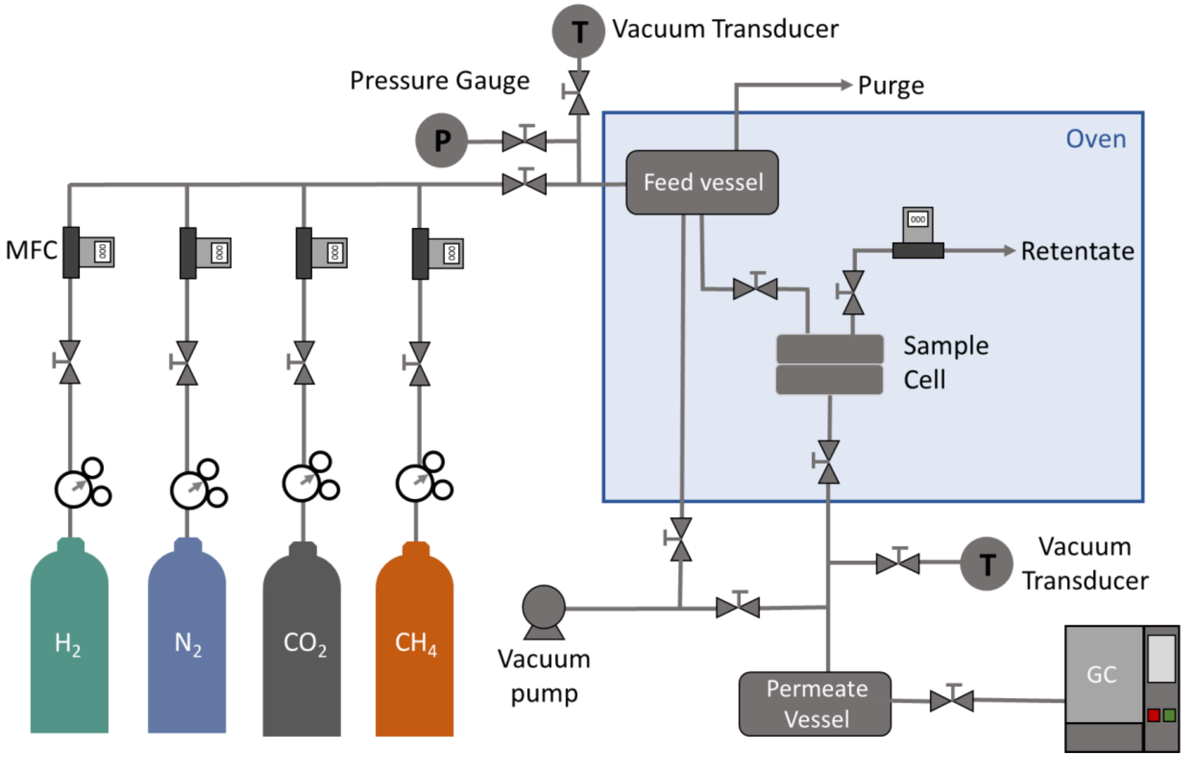

Figure S2. Schematic illustration of the experimental setup for membrane gas permeation tests.

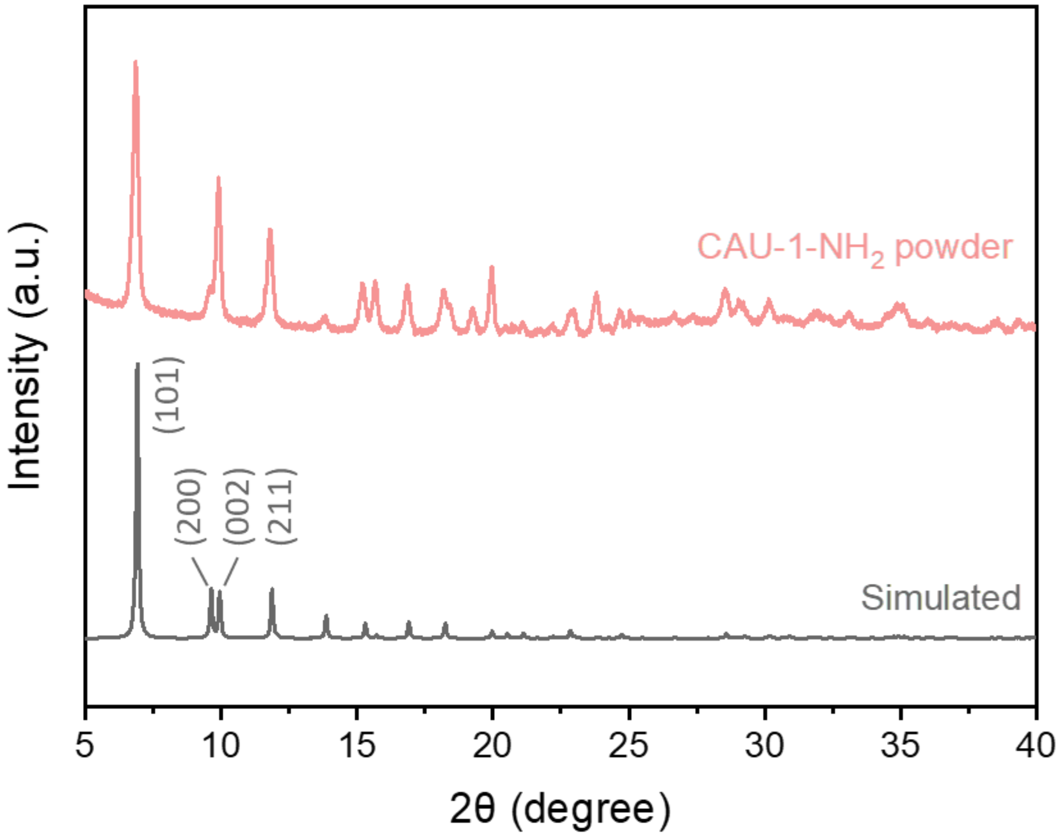

Figure S3. XRD pattern of CAU-1-NH_2 powder compared with the simulated powder diffraction pattern.

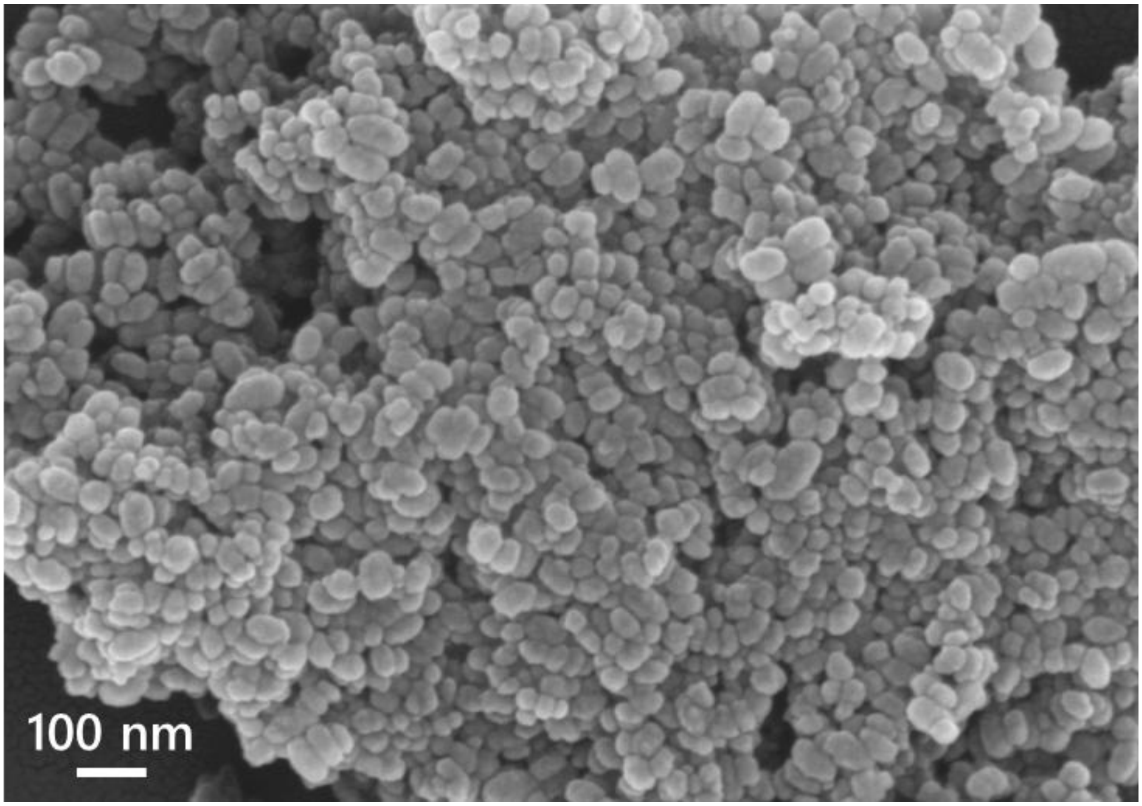

Figure S4. SEM image of the as-synthesized CAU-1-NH_2 powder.

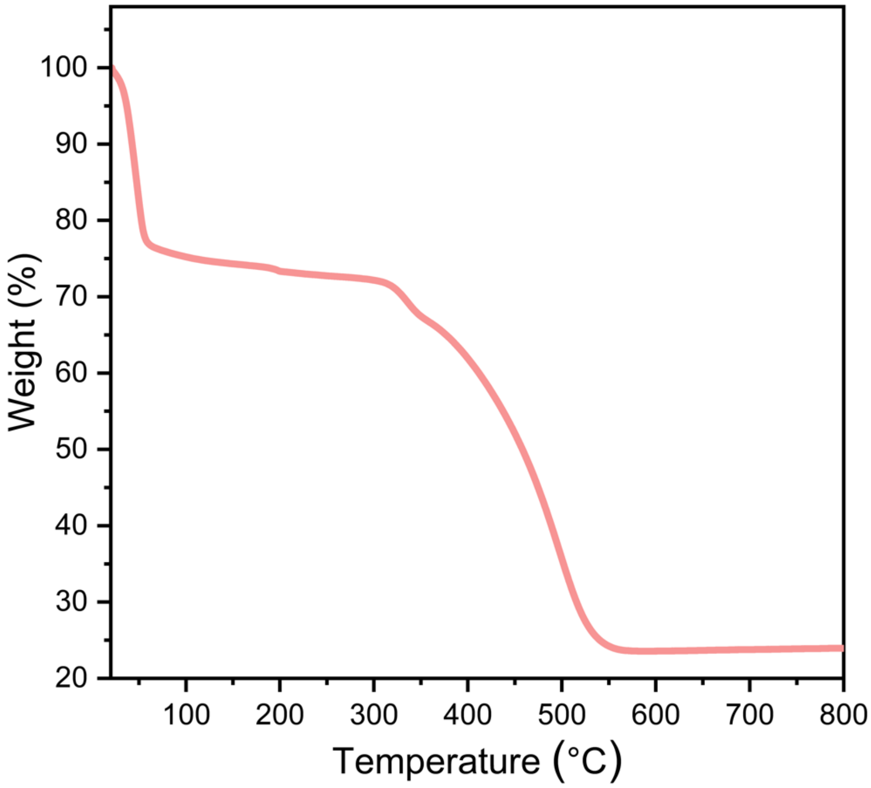

Figure S5. Thermogravimetric analysis (TGA) of the CAU-1-NH_2 powder sample.

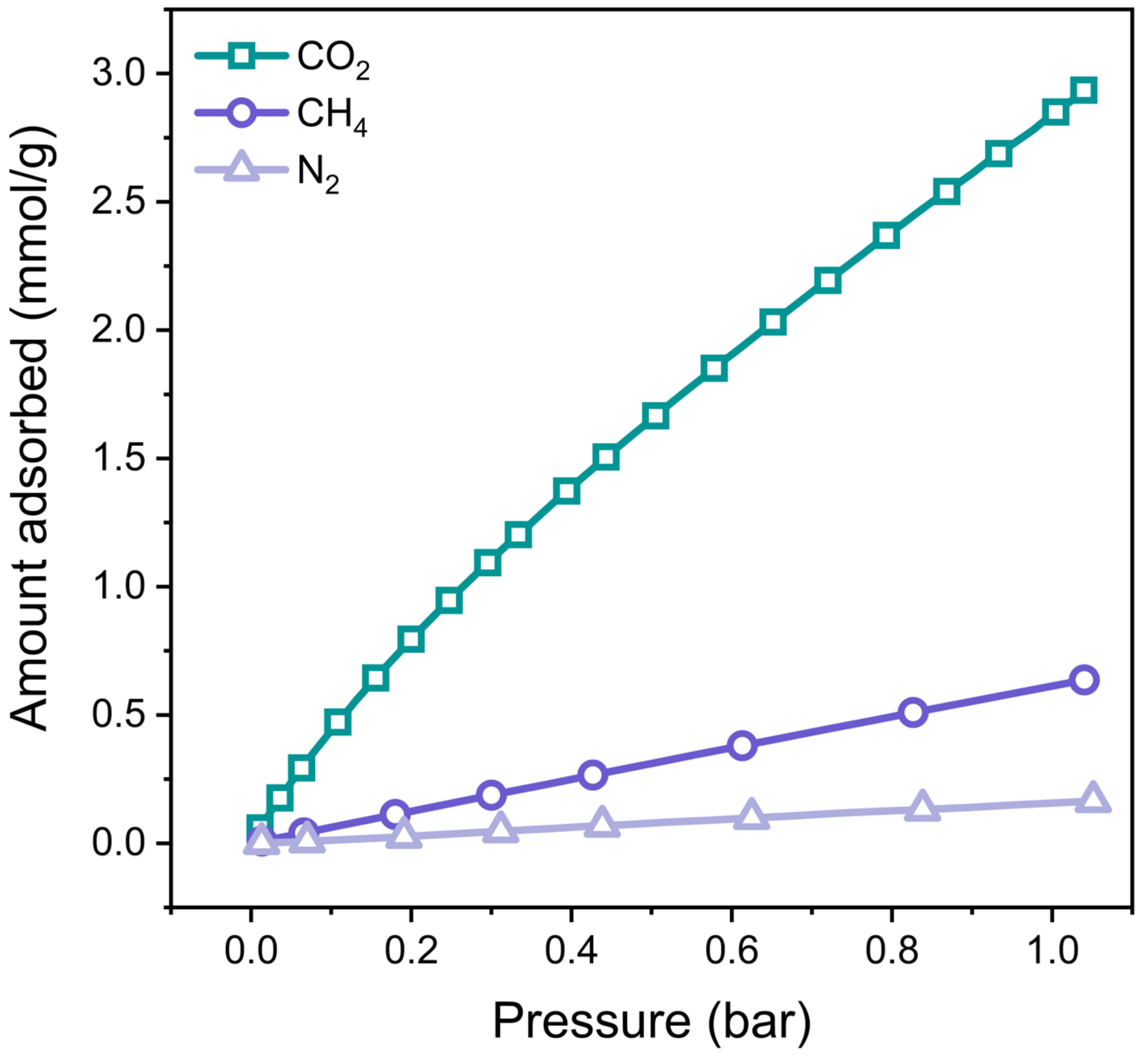

Figure S6. CO_2, N_2, and CH_4 adsorption isotherms of CAU-1-NH_2 measured at 35 °C.

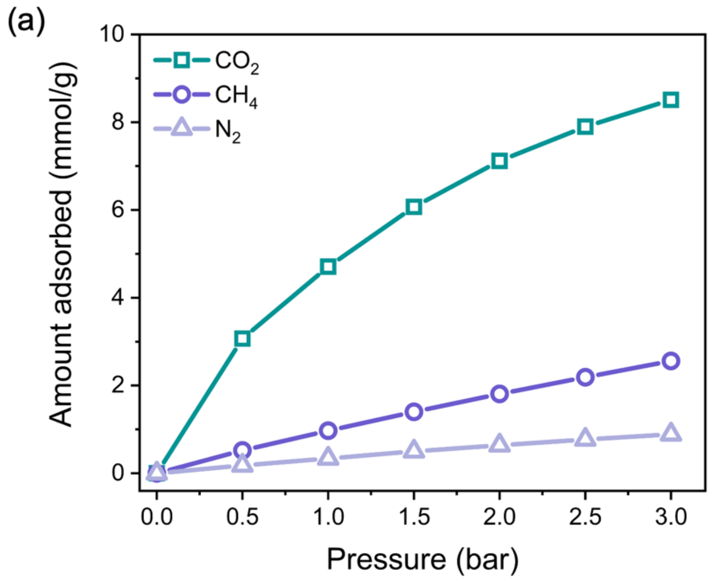

Figure S7. GCMC-simulated adsorption isotherms of CO_2, N_2, and CH_4 in CAU-1-NH_2 at 308 K. (a) Simulations based on the modified crystal structure used in the manuscript, in which residual methyl groups on the metal–oxo nodes were removed and replaced with hydroxyl groups. (b) Simulations based on the structure without removal of the methyl groups. The solubility values reported in the manuscript were calculated using the adsorption data obtained from the structure shown in panel (a).

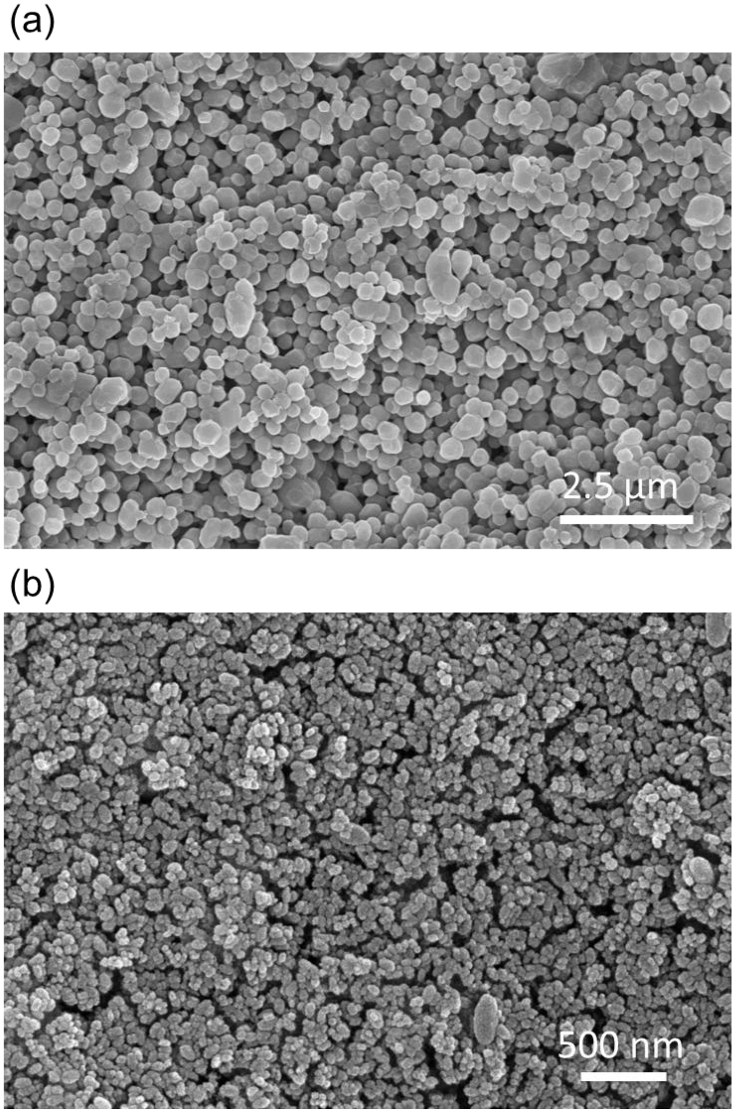

Figure S8. SEM images of (a) the bare α-Al_2O_3 substrate and (b) the substrate after deposition of the CAU-1-NH_2 seed layer.

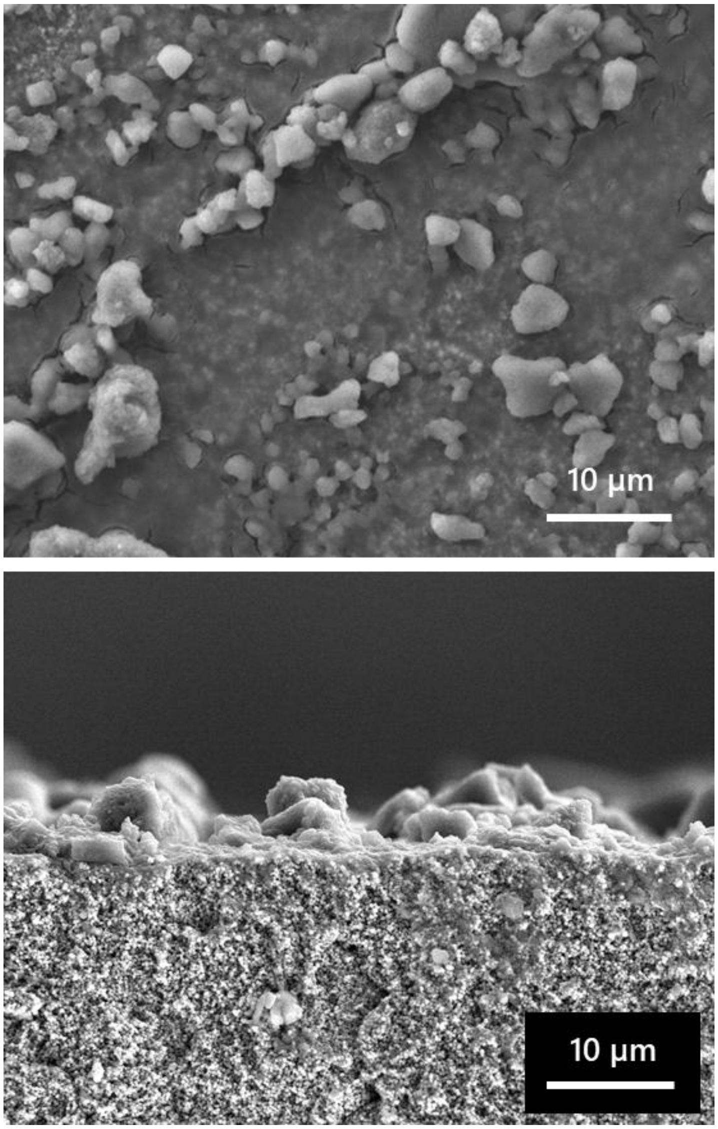

Figure S9. Top-view (top) and cross-sectional (bottom) SEM images of the CAU-1-NH_2(A) membrane.

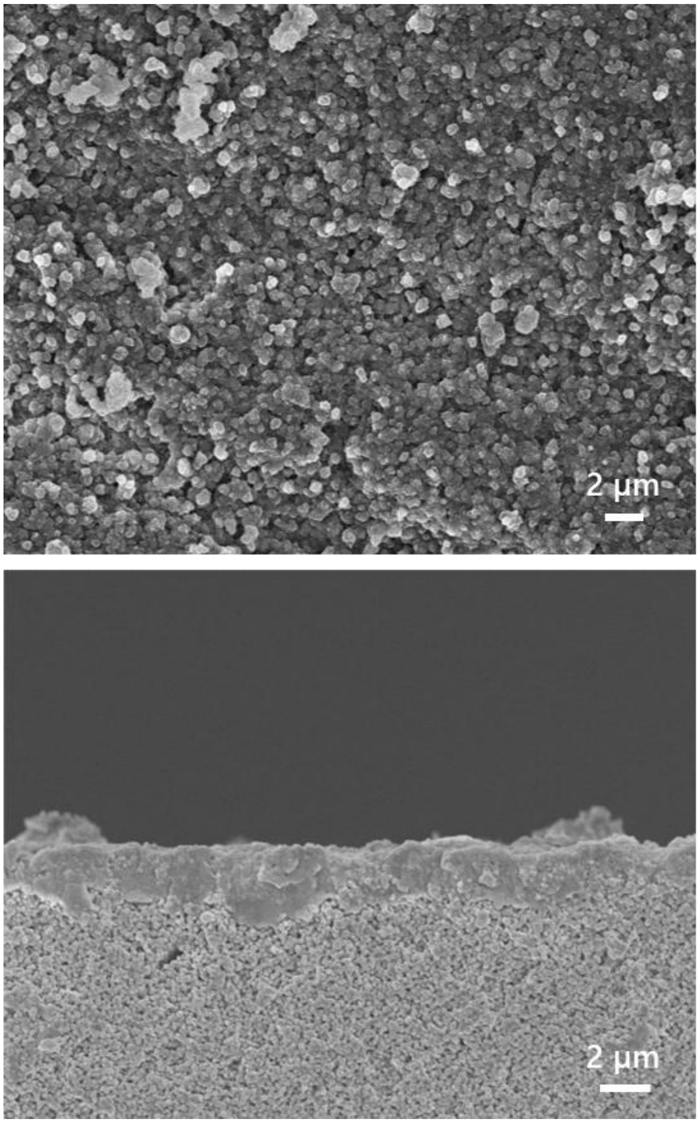

Figure S10. Top-view (top) and cross-sectional (bottom) SEM images of the CAU-1-NH_2(B) membrane.

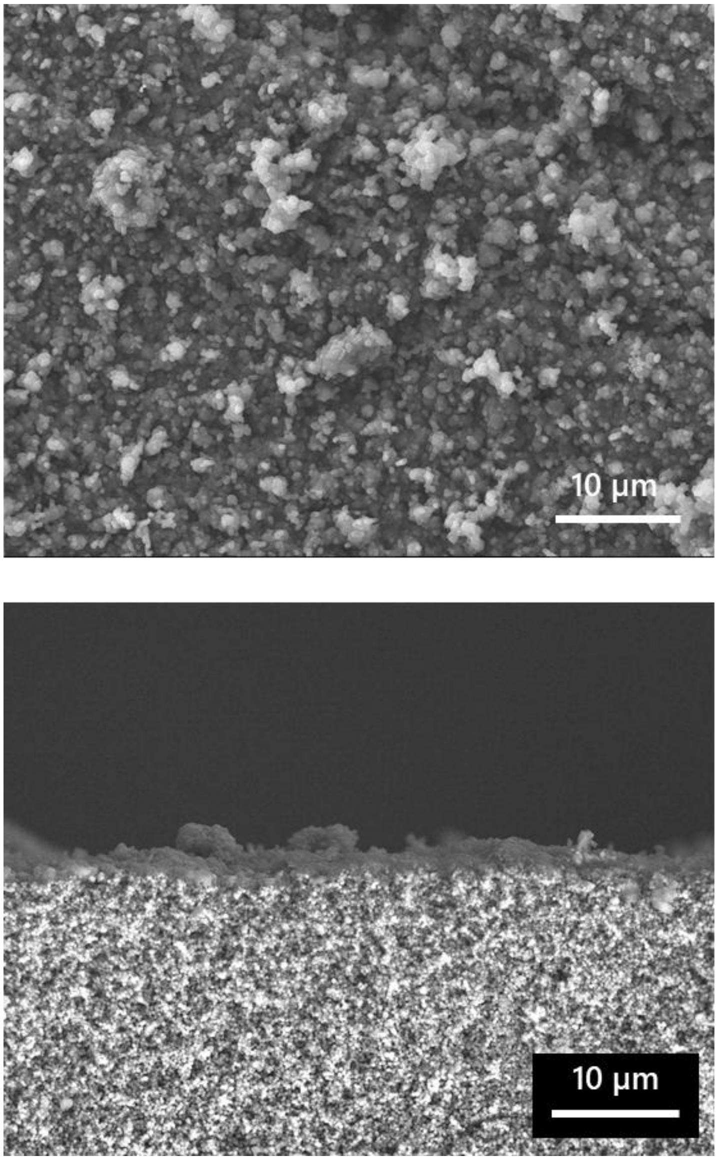

Figure S11. Top-view (top) and cross-sectional (bottom) SEM images of the CAU-1-NH_2(C) membrane.

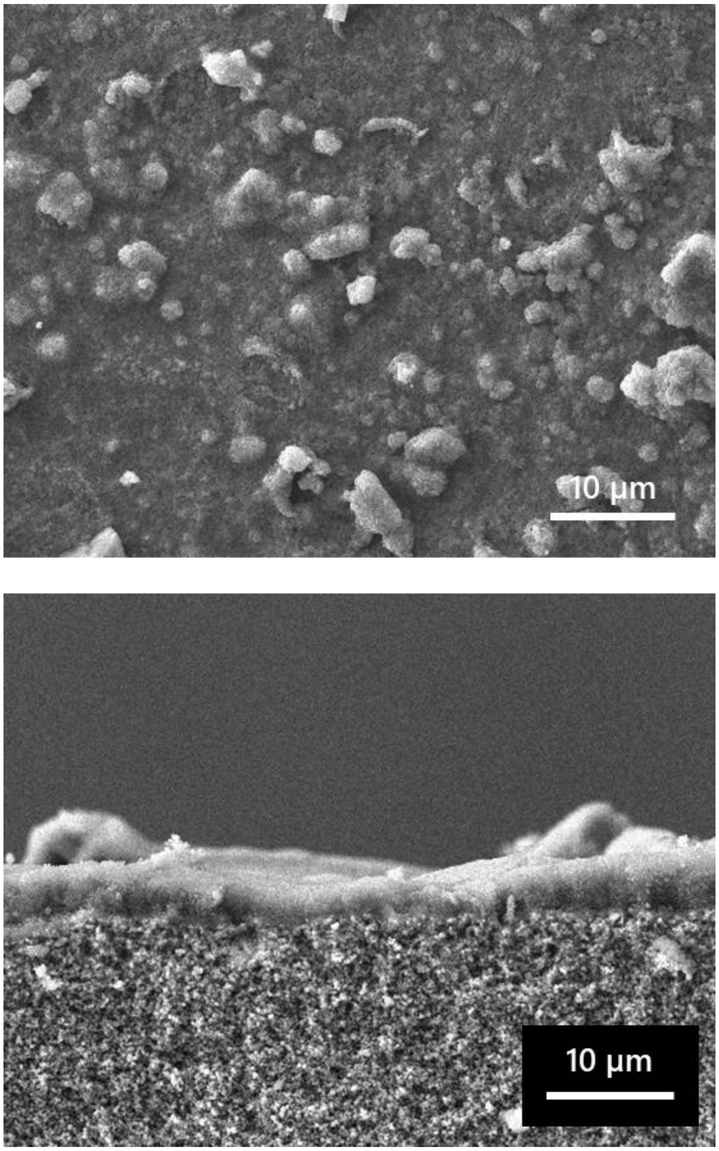

Figure S12. Top-view (top) and cross-sectional (bottom) SEM images of the CAU-1-NH_2(D) membrane.

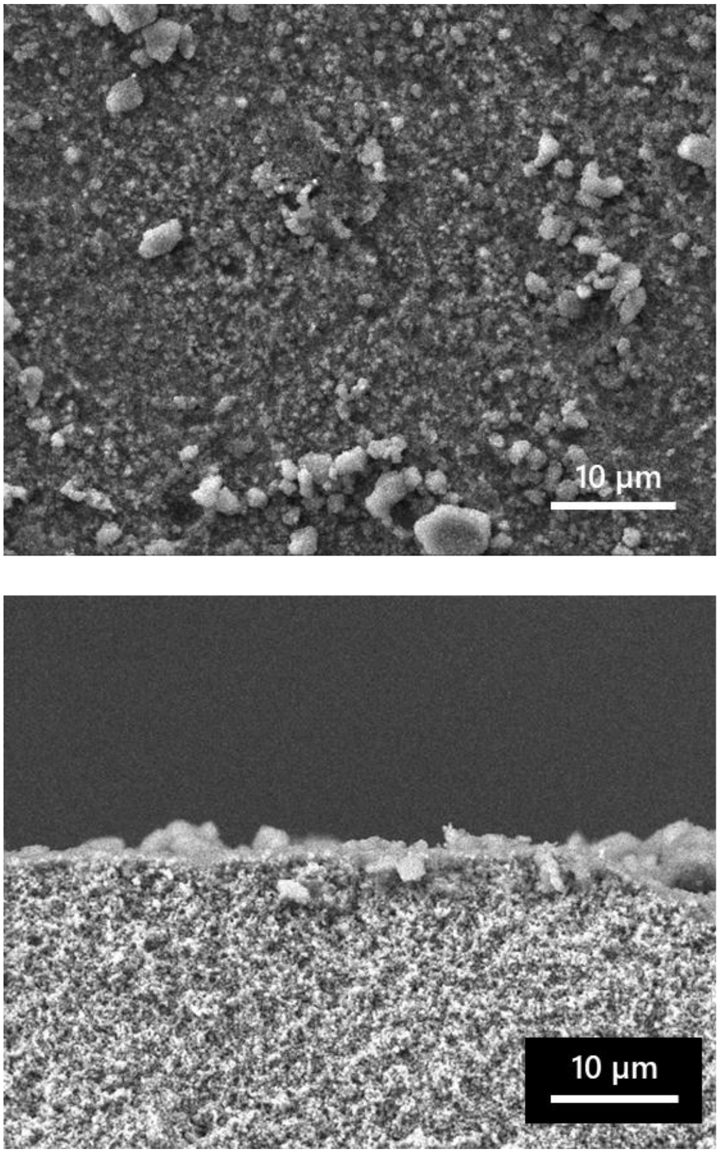

Figure S13. Top-view (top) and cross-sectional (bottom) SEM images of the CAU-1-NH_2(E) membrane.

Table S1. Experimental parameters for the secondary growth of CAU-1-NH_2 membrane variants.

Sample identity	Concentration of $AlCl_3 \cdot 6H_2O$ (M)	Ligand-to-metal molar ratio (-)
A	0.065	0.33
B	0.13	0.33
C	0.26	0.33
D	0.13	1
E	0.13	0.5

Table S2. The CO_2 permeabilities (P_{CO2}) and CO_2/N_2 idea selectivity or mixed-gas separation factors ($\alpha_{CO2/N2}$) of various MOF membranes in this work and based on the literature.

Material	x_{CO2}	Tube or disk	l (μm)	P_{CO2} (Barrer)	Idea selectivity or $\alpha_{CO2/N2}$ (-)	Reference number in the main text
CAU-1-NH_2	Single gas	Disk	3	3.54×10^{2}	19.4	This work
CAU-1-NH_2	0.5	Disk	3	3.47×10^{2}	89.2	This work
CAU-1-NH_2	0.3	Disk	3	4.11×10^{2}	71.5	This work
CAU-1-NH_2	0.1	Disk	3	7.11×10^{2}	59.3	This work
CAU-1-NH_2	0.05	Disk	3	6.85×10^{2}	83.3	This work
ZIF-69	Single gas	Disk	40	1.23×10^{4}	6.3	70
UTSA-280	0.2	Disk	10.0	4.96×10^{1}	42.6	69
CAU-10-H	Single gas	Disk	8.0	5.07×10^{2}	42	51
ZIF-62	Single gas	Disk	25	2.60×10^{3}	34	71
CAU-1	Single gas	Tube	3.0	1.22×10^{4}	15.9	72
Uio-66-ideal	Single gas	Disk	3.5	2.09×10^{3}	31.3	73
MIL-160	0.1	tube	20.4	3.88×10^{3}	178	45
CAU-10-PDC-H(7:3)	Single gas	Disk	10	1.08×10^{3}	25.1	52
MOF-5	0.88	Disk	14	2.09×10^{4}	70	74
Uio-66	0.5	Disk	3.5	2.46×10^{2}	21.4	73
ZIF-8(RHT) -1	Single gas	Disk	0.55	6.57×10^{1}	37.3	75
Sod-ZMOF-1	Single gas	Disk	37.5	7.05×10^{1}	8.7	76

x_{CO2} and l presented in the table, refer to the mole fraction of CO_2 on the feed side and the membrane thickness, respectively, as defined in Eq. 1 and 2 provided in the main text.

Table S3. The CO_2 permeabilities (P_{CO2}) and CO_2/CH_4 idea selectivity or mixed-gas separation factors ($\alpha_{CO2/CH4}$) of various MOF membranes in this work and based on the literature.

Material	x_{CO2}	Tube or disk	l (μm)	P_{CO2} (Barrer)	Idea selectivity or $\alpha_{CO2/CH4}$ (-)	Reference number in the main text
CAU-1-NH_2	Single gas	Disk	3	3.54×10^2	17.6	This work
CAU-1-NH_2	0.5	Disk	3	3.02×10^2	29.1	This work
CAU-1-NH_2	0.3	Disk	3	3.20×10^2	23.1	This work
CAU-1-NH_2	0.1	Disk	3	3.38×10^2	21.8	This work
CAU-1-NH_2	0.05	Disk	3	3.08×10^2	22.3	This work
Kgm-H	0.5	Disk	6	22.0	6.10	77
Kgm-OMe	0.5	Disk	4	4.30	7.80	77
ZIF-69	0.5	Disk	40	1.22×10^4	4.60	70
ZIF-7x-8	Single gas	Disk	0.5	2.25×10^1	25	78
ZIF-8-RHT	0.5	Disk	0.55	77.2	28.8	75
CAU-10-H	0.5	Disk	4	2.24×10^3	50	51
UiO-66	0.5	Disk	20.4	4.36×10^2	9.3	73
UTSA-280	0.2	Disk	10	49.6	42.6	69
CAU-1	Single gas	Tube	2.5	9.86×10^3	14.8	72
ZIF-62	0.50	Disk	70	2.63×10^3	36	71
CAU-10-H	Single gas	Disk	4	5.08×10^2	95	51
CAU-10-PDC-H (7:3)	Single gas	Disk	10	31.7	36.5	52

x_{CO2} and l presented in the table, refer to the mole fraction of CO_2 on the feed side and the membrane thickness, respectively, as defined in Eq. 1 and 2 provided in the main text.
